# Bacterial diversity and predicted enzymatic function in a multipurpose surface water system – from wastewater effluent discharges to drinking water production

**DOI:** 10.1186/s40793-021-00379-w

**Published:** 2021-05-22

**Authors:** Ananda Tiwari, Anna-Maria Hokajärvi, Jorge Santo Domingo, Michael Elk, Balamuralikrishna Jayaprakash, Hodon Ryu, Sallamaari Siponen, Asko Vepsäläinen, Ari Kauppinen, Osmo Puurunen, Aki Artimo, Noora Perkola, Timo Huttula, Ilkka T. Miettinen, Tarja Pitkänen

**Affiliations:** 1 Finnish Institute for Health and Welfare, P.O. Box 95, 70701 Kuopio, Finland; 2 Office of Research and Development, United States Environmental Protection Agency, 26 West Martin Luther King Drive, Cincinnati, OH USA; 3 Pegasus Technical Services, Inc., Cincinnati, OH USA; 4 Present address: Finnish Food Authority, Laboratory and Research Division, Virology Unit, Helsinki, Finland; 5 Turku Region Water Ltd., Turku, Finland; 6 Finnish Environment Institute (SYKE), Latokartanonkaari 11, 00790 Helsinki, Finland; 7 Finnish Environment Institute (SYKE), Survontie 9 A, Jyväskylä, Finland; 8 Faculty of Veterinary Medicine, Department Food Hygiene and Environmental Health, University of Helsinki, Helsinki, Finland

**Keywords:** Bacterial communities, Sewage effluent, Surface water, Seasonal effects, Predicted biological function, 16S rRNA amplicon sequencing

## Abstract

**Background:**

Rivers and lakes are used for multiple purposes such as for drinking water (DW) production, recreation, and as recipients of wastewater from various sources. The deterioration of surface water quality with wastewater is well-known, but less is known about the bacterial community dynamics in the affected surface waters. Understanding the bacterial community characteristics —from the source of contamination, through the watershed to the DW production process—may help safeguard human health and the environment.

**Results:**

The spatial and seasonal dynamics of bacterial communities, their predicted functions, and potential health-related bacterial (PHRB) reads within the Kokemäenjoki River watershed in southwest Finland were analyzed with the 16S rRNA-gene amplicon sequencing method. Water samples were collected from various sampling points of the watershed, from its major pollution sources (sewage influent and effluent, industrial effluent, mine runoff) and different stages of the DW treatment process (pre-treatment, groundwater observation well, DW production well) by using the river water as raw water with an artificial groundwater recharge (AGR).

The beta-diversity analysis revealed that bacterial communities were highly varied among sample groups (R = 0.92, *p* <  0.001, ANOSIM). The species richness and evenness indices were highest in surface water (Chao1; 920 ± 10) among sample groups and gradually decreased during the DW treatment process (DW production well; Chao1: 320 ± 20). Although the phylum *Proteobacteria* was omnipresent, its relative abundance was higher in sewage and industrial effluents (66–80%) than in surface water (55%). Phyla *Firmicutes* and *Fusobacteria* were only detected in sewage samples. *Actinobacteria* was more abundant in the surface water (≥13%) than in other groups (≤3%). *Acidobacteria* was more abundant in the DW treatment process (≥13%) than in others (≤2%). In total, the share of PHRB reads was higher in sewage and surface water than in the DW treatment samples. The seasonal effect in bacterial communities was observed only on surface water samples, with the lowest diversity during summer.

**Conclusions:**

The low bacterial diversity and absence of PHRB read in the DW samples indicate AGR can produce biologically stable and microbiologically safe drinking water. Furthermore, the significantly different bacterial communities at the pollution sources compared to surface water and DW samples highlight the importance of effective wastewater treatment for protecting the environment and human health.

**Supplementary Information:**

The online version contains supplementary material available at 10.1186/s40793-021-00379-w.

## Introduction

Assuring an adequate supply of high-quality raw water for the production of drinking water (DW) is a challenge worldwide. In most cases, surface waters such as rivers and lakes near cities fulfill the demand for raw water [2]. However, anthropogenic sources of pollution via discharges of treated municipal and industrial effluents often pose a threat to the surface water quality. Despite the highly developed wastewater treatment techniques, not all pollutants are sufficiently removed [22]. Occasionally, raw sewage from combined sewer overflows may also contaminate river water [19]. In addition, diffuse sources of pollution—such as runoff from agricultural land, forest areas, and urban flow during heavy rains and snowmelt may—deteriorate surface water quality. Overall, the protection of raw water quality from multiple sources of pollution is a critical task for maintaining environmental health. Besides surface waters, groundwater can be a good source of high-quality raw water for DW production. However, the high drinking water demand in some geographical locations may require the use of artificial groundwater recharge (AGR) [29]. In Finland, for example, AGR and groundwater together fulfill about 60% of the raw water demand for DW production, and the rest of the demand is fulfilled from surface waters [2].

Bacterial communities play a central role in aquatic ecosystems and can be affected by various ecological factors in water such as temperature and light conditions, UV radiation, pH, the concentrations of available oxygen, nitrogen, phosphorous, and metal ions [49], the presence of biodegradable pollutants [52], predator interactions and the presence of bacteriophages [33], and land-use patterns of the catchment area [48]. Furthermore, the bacteria introduced from pollutant sources may shape both the taxonomic and functional diversity of the recipient water [26, 52]. While many of the ecological factors may differ depending on geographical location, these may also vary even seasonally at a single location [54], affecting the rate of photosynthesis and ecosystem productivity. Moreover, due to the unidirectional flow of water, ecological factors within a river ecosystem can be almost unique.

Recently, many bacterial community studies have used high-throughput 16S ribosomal RNA gene sequencing analysis [55], including studies in engineered water systems (i.e., drinking water systems) [17, 27] and natural aquatic ecosystems [30, 35, 48]. The 16S rRNA gene amplicon analysis is the most often used to describe the composition of bacterial communities, but it may also provide information about the presence of PHRB [28, 53] and may be used to predict the enzymatic function of aquatic bacterial communities [1, 32, 34]. However, comprehensive information about how the bacterial community changes from sources of contamination to surface waters and into drinking water production is lacking, especially in regards to boreal regions.

The aim of the study was to find out if (a) the bacterial community structure and function—specifically diversity, taxonomy, predicted enzymatic function, and PHRB—change significantly from the sources of contamination through the surface water to drinking water production; (b) the AGR process can produce biologically stable and microbiologically safe drinking water. To reach the study aims, water samples were systematically collected in each season (autumn, winter, spring, and summer) in two consecutive years from a surface water ecosystem consisting of lakes and rivers, its major point pollutant sources all the way to drinking water production with the AGR process, a path rarely followed before. Then, bacterial diversity, taxonomy, predicted enzymatic functions, and changes in the read abundance of potential health-related bacteria (PHRB) over time and sites were evaluated with the 16S rRNA gene sequencing method.

## Materials and methods

### Description of the study area

The study sites were in the Kokemäenjoki River watershed in the southwestern part of Finland (Supplemental Table S1, [19, 44]). The Nordic conditions of the study area consist of four distinct seasons with a high variation in daylight hours, temperature, and precipitation (Supplemental Table S2). The natural discharge in Finnish rivers is usually highest in the spring and early summer due to snowmelt, despite this period having the lowest mean precipitation.

The Kokemäenjoki River flows from Lake Pyhäjärvi near the city of Nokia, towards the southwest coast of Finland. It drains water from diverse sources such as treated municipal and industrial sewage discharges and runoff from urban areas, mines, agricultural fields, and forest areas. The river water is used for bathing and recreational purposes and serves as raw water for DW production (23 million m^3^ / year) in the Turku region (southwest part of Finland) [19, 29]. The AGR technique is used for the drinking water production process. Initially, the raw water from the river is pretreated using sieving, dissolved air flotation, and sand filtration prior to infiltration into the sand/gravel esker aquifer located in the Virttaankangas managed aquifer area [29]. The AGR production plant consists of seven infiltration areas, each having two to four infiltration ponds. The average residence time of the infiltrated water in the esker aquifer is 4 months, and the water quality is monitored from groundwater (GW) observation wells. Potable water is pumped from the DW production wells and supplied to consumers after UV and chloramine disinfection.

A total of seven municipal wastewater treatment plants (WWTPs) discharging treated wastewater effluents into the Kokemäenjoki watershed were included in the study: Rahola and Viinikanlahti in the city of Tampere; Kullaanvuori and Siuro in the city of Nokia; and Mouhijärvi, Vammala, and Äetsä in the city of Sastamala. Viinikanlahti is the largest WWTP of the study, serving about 200,000 inhabitants, and Mouhijärvi is the smallest, serving about 1300 inhabitants [2, 19]. All these WWTPs use primary and secondary treatment processes, including screening, grit removal, and ferric salt addition followed by conventional activated sludge treatment with the addition of flocculants. Industrial effluents were collected from two industrial treatment plants prior to discharge into the river. The mine runoff water was collected from a mining area discharge.

### Water samples

A total of 243 water samples were collected from 30 sampling locations in each season for 2 years from the Kokemäenjoki watershed, its point sources of pollution, and the DW production process (Table 1). Surface water samples were collected from 15 sampling locations, of which two were in the lake region, four in tributary rivers, and nine in the main river.

**Table 1:** Summary of water samples collected in 2012–2014

Site number	Sampling location	Number of samples^a^
**Municipal sewage effluent (*n*** **= 57)**		
1	Tampere, Viinikanlahti	8 (1_b_)
2	Tampere, Rahola	8
3	Nokia, Kullaanvuori	8 (1_b_)
4	Nokia, Siuro	8 (1_b_)
5	Sastamala, Mouhijärvi	8 (1_b_)
6	Sastamala, Vammala	9
7	Sastamala, Äetsä	8 (1_b_)
**Municipal sewage influent (*n*** **= 7)**		
1	From the sewage effluent sampling sites (1 to 7)	7
**Industrial effluent and mine runoff (*n*** **= 20)**		
1	Industry I	7 (1_b_)
2	Industry II	9
3	Mine	4
**Surface water (*n*** **= 119)**		
1	Ratinanvuolle (tributary)	8 (1_b_)
2	Pyhäjärvi, depth 1 m (lake region)	8
	Pyhäjärvi, depth 10 m (lake region)	5
	Pyhäjärvi, depth 40 m (lake region)	5
3	Rajasaari (lake region)	4
4	Sotkanvirta (tributary)	8 (1_b_)
5	Nokiankoski (upstream)	8
6	Nokiankoski (downstream)	8 (1_b_)
7	Siuronkoski (tributary)	8
8	Hiedanvuolle	8
9	Rautavesi	8
10	Liekovesi	8 (1_b_)
11	Keikyä	8
12	Karhiniemi (raw water)	5
13	Karhiniemi	8
14	Loimijoki (tributary)	8
15	Kojo, Kolsi	4
**Drinking water treatment process with AGR (*n*** **= 40)**		
1	Pretreated water	11
2	Groundwater observation well 1	9
3	Groundwater observation well 2	9 (2_b_)
4	Production well	11 (1_b_)
**Total [*N*** **= 243]** (13_b_)		

^a^ *n* = total number of samples in each sample group. Drinking water treatment samples were collected in each season between autumn 2012 and autumn 2014. The rest of the sample groups were sampled from autumn 2012 to spring 2014 except the municipal sewage influent sample, which was collected only once during the autumn 2013 sampling campaign. The samples marked as _b_ had low sequence reads and were not included for further analysis. The total number of samples remaining for further analysis was 230

All surface water samples were collected with a grab sampler from one meter below the surface except at Site 2 (located on the deepest region of the lake), where the samples were collected from one meter, 10 m, and 40 m below the surface to assess if sampling depth had an effect on lake bacterial communities. Also, groundwater samples of infiltrated water from observation and production wells and wastewater samples from WWTPs were collected as grab samples. The DW production well samples were collected before adding any disinfection, as the current study followed the path from surface water to the DW production well. Water samples were transported in coolers to the laboratory of the Finnish Institute for Health and Welfare (Kuopio, Finland) and processed within 24 h.

### DNA extraction, amplification, and sequencing

Collected samples were stored by filtering the samples of surface water (75 ml – 400 ml), sewage effluent (50 ml – 100 ml), and groundwater (500 ml – 1000 ml) onto nylon membranes with a pore size of 0.2 μm (N66, Ultipor, Pall Corporation, Ann Arbor, Michigan, USA). Immediately after filtration, the membranes were treated with RNAlater (Qiagen, Hilden, Germany) and kept at 4 °C overnight before freezing at − 75 °C [44].

DNA was extracted from stored filters, which were first transferred to microcentrifuge tubes with acid-washed DNase and RNase free glass beads (Mo Bio Laboratories, Inc., Carlsbad, California, USA). Storage tubes containing RNAlater were centrifuged for 3 min at maximum speed, and the pellet was resuspended with 500 μl lysis buffer (Buffer RLT Plus (Qiagen, Hilden, Germany) containing β-mercaptoethanol (Sigma-Aldrich Co., St. Louis, MO) and added to the microcentrifuge tube containing the filter. The tubes were then bead-beated for 40 s at maximum speed (Mini-Bead-Beater, Biospec Products, Inc., Bartlesville, Oklahoma, USA) and centrifuged 3 min at maximum speed. The DNA fraction was extracted using AllPrep DNA/RNA Mini Kit (Qiagen GmbH, Germany) following the manufacturer’s protocol.

The DNA extracts were shipped on dry ice to the laboratory of the United States Environmental Protection Agency (Cincinnati, Ohio) for community sequencing, as previously described [5, 27]. Specifically, DNA extracts were used as templates for 16S rRNA gene metabarcoding primers. We used barcoded primers 515F and 806R [10] to construct 16S rRNA gene sequence libraries for each sample tested. The PCR assays used for the sequencing libraries were performed in 25-μl volumes using the Ex Taq kit (TaKaRa) with 200 nM concentrations (each) of the forward and reverse primers and 2 μl of DNA extracts and using the following cycling conditions: an initial 5 min denaturing step at 95 °C, followed by 35 cycles at 95 °C for 45 s, 50 °C for 60 s, and 72 °C for 90 s, and a final elongation step at 72 °C for 10 min. Each barcode corresponded to an eight-base sequence unique to each sample. Amplicons were visualized on an agarose gel to confirm product sizes, and aliquots of each amplicon of the expected size were pooled and sequenced using an Illumina MiSeq sequencer and 250-bp paired-end kits at the Cincinnati Children’s Hospital DNA Core facility.

### Sequence data processing and bacterial community analysis

The fastq files with forward and reverse reads of bacterial 16S rRNA gene obtained from 300 bp paired-end Illumina MiSeq sequencing were merged with Flash software version 1.1 [37]. Trimming of bad quality reads, removal of primer and adapter sequences, and removal of ambiguous and short-length sequences was done using Quantitative Insights Into Microbial Ecology (QIIME) bioinformatics pipeline version 1.8.0 [9] with split_libraries_fastq.py script. Chimeras were removed with the usearch61 [15] method using the identify_chimeric_seqs.py script. After chimera removal, the preprocessed reads were aligned with the Greengenes database [12] version 13_8 [38] and sorted with > 97% similarity alignment with PyNAST [8] into operational taxonomic units (OTUs) using the closed reference OTU picking approach with the UCLUST algorithm [16]. An attempt to filter out the mitochondrial and chloroplast reads was made along with the singleton OTUs.

The bacterial communities were further analyzed with MicrobiomeAnalyst [13]. A total of 230 samples out of 243 samples had total read counts higher than the rarefaction value (i.e., 4860) and were used for further study. Bacterial communities in the surface water sample subgroups originating from the lake region, watershed tributaries, and the main river region demonstrated no distinct beta-diversity values (R = 0.10, *p* <  0.002, ANOSIM; Table 2), and therefore these samples were handled as a single group in the further analysis. Furthermore, the samples collected from three different depths in the Pyhäjärvi Lake sampling location (Site 2) were handled together as one sampling site, although the beta-diversity analysis was not statistically significant (R = 0.07, *p* <  0.196, ANOSIM; Table 2).

**Table 2:** Significance of the bacterial community differences between sample types and sampling seasons

Samples included	Experimental grouping	R^b^	***p***-value
All samples	Sample groups^a^	**0.92**	< 0.001
	Seasons	0.04	< 0.003
Sewage	Effluent and Influents	**0.34**	< 0.001
Sewage effluent	Seasons	0.20	< 0.001
Industrial effluents	Industry I and Industry II	**0.67**	< 0.001
	Seasons	−0.09	< 0.737^a^
Surface Water	Lake region, tributaries, and main river	0.10	< 0.002
	Seasons	**0.38**	< 0.001
	Sampling depth 1 m, 10 m, and 40 m in Site 2 of surface water (Pyhäjärvi)	0.07	< 0.196^a^
Treated samples	Pretreated water and AGR process water (combining groundwater observation well and production well samples)	**0.96**	< 0.001
	Seasons	−0.06	< 0.970^a^

^a^ Ordination method: PCoA, distance method: Bray-Curtis index, taxonomic level: OTU, statistical method: analysis of group similarities (ANOSIM). Sample groups are presented in Table 1 and Fig. 2. ^b^Evaluation criteria: (a) 0.75 < R < 1, highly separate; (b) 0.5 < R < 0.75, separate; (c) 0.25 < R < 0.5, separate with some overlap; (d) 0.1 < R < 0.25, similar with some differences; (e) R < 0.1, similar [46]. The experimental groups having separate and significantly different bacterial communities are highlighted **in bold and underlined**. Sample groups showing *p-*value with * had low sample numbers, so they did not have sufficient itineration during analysis

Herein, the core microbiome refers to the set of taxa that have higher relative abundance above a given abundance threshold. The core bacterial communities were calculated with MicrobiomeAnalyst in a way that an OTU with a relative read abundance of more than 0.01% of total reads in a sample was defined as the core community of that sample. The core OTUs detected in more than 20% of samples (sample prevalence > 20%) in a studied group was reported as the core community of that sample group. The core bacterial OTUs were classified into the deepest possible taxonomic level with the Greengenes OTU annotation library.

The predicted enzymatic function of bacterial communities was calculated with 16S rRNA gene sequence library-based OTUs with PICRUSt [34] with MicrobiomeAnalyst. The PICRUSt produced a Kyoto encyclopedia of genes and a genomes orthology (KEGG Orthology) matrix. The KEGG Orthology (KO) refers to groups linked to molecular functions represented in the KEGG database (www.genome.jp/kegg). The KO analysis for 230 water samples was performed as follows: (a) a total KO list was obtained from MicrobiomeAnalyst; (b) for each ortholog, the KO with the highest number was identified; and (c) all orthologs were re-arranged to select only the orthologs that had the highest value of 500 or more in Step b for the further identification of KEGG pathways. The total number of each functional category was calculated with a simple sum [13] and compared on different sample groups and seasons.

The list of screened potential health-related bacteria (PHRB) is shown in Supplemental Table S3. The detection frequency of total reads of each PHRB genus was analyzed within the whole data (*n* = 230 retained after removing 13 low read samples) and in addition for each sample group in different seasons of the year. The detection frequency of tentative PHRB genus *Arcobacter* spp. [43] was high (92% in the whole data), and the genus had high relative abundance in sewage influent (44% of total reads), sewage effluent (11% of total reads), and mine runoff (4% of total reads). Therefore, the genus was not included in the PHRB analysis intended for rare species.

### Statistics

Alpha-diversity was calculated by MicrobiomeAnalyst using OTU counts, observed species index, Chao1 index, and abundance-based coverage estimator (ACE) as taxonomic richness indices [39]. The observed species index measures the total numbers of possible unique species. The Chao1 index, a non-parametric measure, analyzes the ratio of singleton reads (*n* = 1) to doubleton reads (*n* = 2) and assigns more weight to the rare species [39]. Shannon and Simpson diversity indices were used as measures for taxonomic richness and evenness.

Analysis of similarity (ANOSIM) method from MicrobiomeAnalyst was used for non-metric multi-dimensional scaling (NMDS) of beta-diversity with the Bray-Curtis index distance method. The comparison was made following the criteria reported by Ramette [46]: (a) 0.75 < R < 1, highly separate; (b) 0.5 < R < 0.75, separate; (c) 0.25 < R < 0.5, separate with some overlap; (d) 0.1 < R < 0.25, similar with some differences; and (e) R < 0.1, similar.

The significance of the difference between the sample groups and seasons was studied with the Kruskal-Wallis test followed by the Dunn-Bonferroni post-hoc test in IBM SPSS Statistics for Windows version 25. The *p*-values < 0.05 were considered statistically significant. The normality of data was tested by the Shapiro-Wilk test. Because many PHRB reads had zero read counts in samples, the bacterial read counts were log-transformed by (Log_10_ N + 1) before statistical analysis. Since the data were highly skewed even after the logarithmic transformation, the 95% confidence limit of median reads of PHRB was calculated with the following equations [7] from PHRB read counts arranged in ascending order:

$$ Lower\kern0.17em confidence\kern0.17em limit={\left(\frac{n}{2}-1.96\frac{\sqrt{n}}{2}\right)}^{th}\; observation $$

$$ Upper\kern0.17em confidence\kern0.17em limit={\left(1+\frac{n}{2}+1.96\frac{\sqrt{n}}{2}\right)}^{th}\; observation $$

where n is the total number of observations in the studied sample group.

## Results

A total of 6,052,510 high-quality reads were obtained after sequencing, aligning, and chimera removal. The average read count per sample was 26,320 (median 23,190), ranging from 4860 to 65,480 reads. The rarefaction curves of all sample groups are presented in the Supplemental Material (Figure S1). The average read counts from the drinking water treatment process samples, the GW observation well, and the production well were lower than from surface water, sewage influent, sewage effluent, and industrial effluents (Table 3; *p* < 0.001, Kruskal-Wallis). No significant changes were observed in the average read counts between the seasons of the year (*p* = 0.51, Kruskal-Wallis).

**Table 3:** Geometric mean (±standard error) of sequencing reads and alpha-diversity indices in sample groups

Parameter	Sewage influent	Sewage effluent	Industrial effluent	Mine runoff	Surface water	Pre-treated	GW observation well	Production well
Reads	34,300 ± 4300	26,200 ± 1800	20,600 ± 3700	13,300 ± 2200	24,200 ± 1100	32,700 ± 5400	15,900 ± 2600	11,300 ± 2100
OTUs	700 ± 40	800 ± 20	290 ± 40	1120 ± 50	890 ± 20	630 ± 40	470 ± 30	370 ± 10
Observed	490 ± 30	580 ± 10	170 ± 20	640 ± 20	730 ± 10	530 ± 30	320 ± 30	250 ± 10
ACE	940 ± 60	1080 ± 30	510 ± 40	1490 ± 60	1230 ± 20	880 ± 60	590 ± 40	480 ± 20
Chao1	630 ± 40	720 ± 10	300 ± 30	810 ± 30	920 ± 10	710 ± 40	410 ± 30	320 ± 20
Shannon	3.96 ± 0.15	4.15 ± 0.07	2.74 ± 0.26	5.35 ± 0.22	4.91 ± 0.02	3.99 ± 0.10	4.60 ± 0.07	4.15 ± 0.09
Simpson	0.91 ± 0.01	0.92 ± 0.01	0.78 ± 0.03	0.97 ± 0.01	0.98 ± 0.00	0.94 ± 0.01	0.96 ± 0.00	0.93 ± 0.01

### Taxonomic diversity (alpha-diversity)

A total of 3823 OTUs were observed in the samples included in the analysis (n = 230). Specifically, 3388, 2554, 962, 1269, and 1711 OTUs were recorded for surface water (n = 115), municipal sewage (*n* = 59), industrial effluents (*n* = 15), mine runoff (n = 4), and drinking water (*n* = 37), respectively. The geometric mean (± standard error) of diversity indices in each sample group is shown in Table 3, and the box-plot comparison is shown in supplemental Figures S2 and S3. The geometric mean of richness indices increased after sewage treatment (i.e., influent vs. effluent), while the indices were lower in industrial effluent than in municipal sewage and mine runoff samples (Table 3). All richness indices were gradually reduced from surface water (Chao1: 920 ± 10) to the DW production well (Chao1: 320 ± 20) during the drinking water treatment process (Table 3, supplemental Figures S2 and S3). The taxonomic richness and evenness indices had a positive correlation to each other (Supplemental Table S4).

Among the surface water sample subgroups (tributary river, lake, and river water), river water had a significantly higher Shannon diversity index (*p* = 0.002, Kruskal-Wallis) and Chao 1 Index (*p <* 0.001, Kruskal-Wallis) (Supplemental Figure S4 B). The season of the year affected taxonomic diversity indices only within the surface water samples (Table S5, supplemental Figures S5 and S6). The alpha-diversity of the bacterial community was significantly lower in the summer than in the other seasons (Chao1; autumn: 950 ± 20, winter: 990 ± 30, spring: 960 ± 30, summer: 790 ± 20; *p* < 0.001, Kruskal-Wallis).

### Bacterial community variation in the sample groups (beta-diversity)

**Fig. 1:**
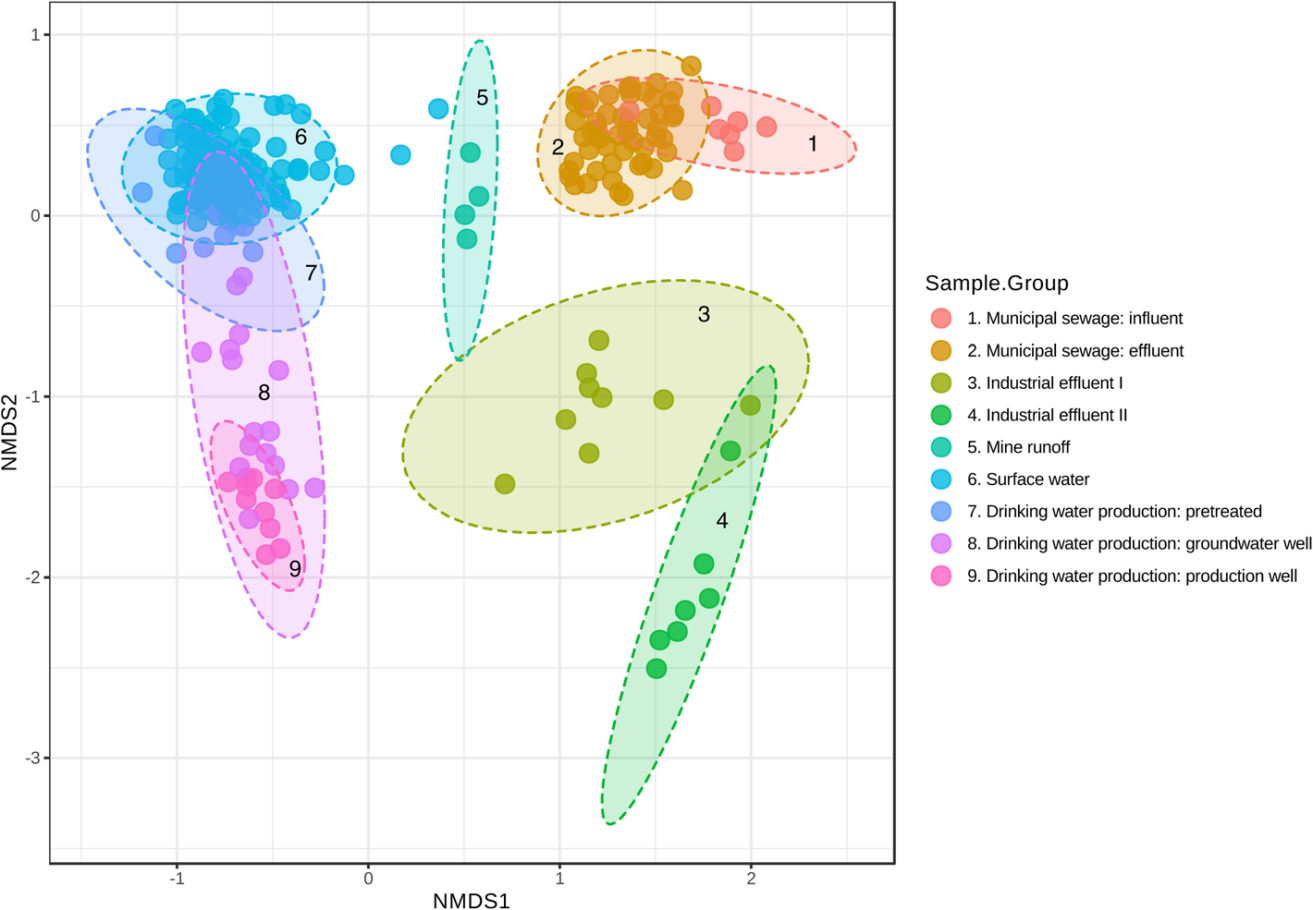
Nonmetric multidimensional scaling and analysis of dissimilarities between bacterial communities in sampling groups [ANOSIM] R = 0.92; *p*-value < 0.001 [NMDS] Stress = 0.11567. Ordination method: NMDS, distance method: Bray-Curtis index, taxonomic level: OTU, statistical method: analysis of groups similarities (ANOSIM)

Based on the nonmetric multidimensional scaling (NMDS) and the beta-diversity of bacterial taxa, five major clusters were noted—municipal sewage, industrial effluent, mine runoff, surface water, and the samples from the AGR production process (Fig. 1). Furthermore, within municipal sewage, there were significant differences in bacterial communities between effluent and influent (R = 0.34, *p* < 0.010, ANOSIM)—and within industrial effluent—between Industry I and Industry II (R = 0.67, p < 0.010, ANOSIM; Table 2). Bacterial communities were similar within the surface water sample subgroups (tributary river, lake, and river water, R = 0.10, *p* < 0.002; ANOSIM; Supplemental Figure S4 A). Among the three different drinking water production steps, there were considerable differences between the pretreated samples and the AGR samples (R = 0.96, *p* < 0.010; ANOSIM). Following the water flow in the AGR process, the sample collected from the GW Observation Well I was closer to the pretreated samples, while the sample collected from GW Observation Well II overlapped with the production well samples (Fig. 1). However, although the samples from GW Observation Well I seemed to overlap with the pretreated samples in the two-dimensional plots (Fig. 1), these samples were highly separate on a three-dimensional scale (data not shown).

### Bacterial community at the phylum, class, and family levels

A total of 38 bacterial phyla from the Greengenes database were identified via high-throughput 16S rRNA gene sequence analysis. All these phyla were present in surface water samples, but only 25, 30, 32, 20, and 22 phyla were recorded from industrial effluent (both I and II), mine runoff, sewage (both effluent and influent), pretreated water, and AGR samples, respectively. About 40% of phyla from each sample group had a read contribution of more than 0.1% out of all reads in that sample group. Hereafter, we report bacterial community composition at the phylum, class, and family levels.

**Fig. 2:**
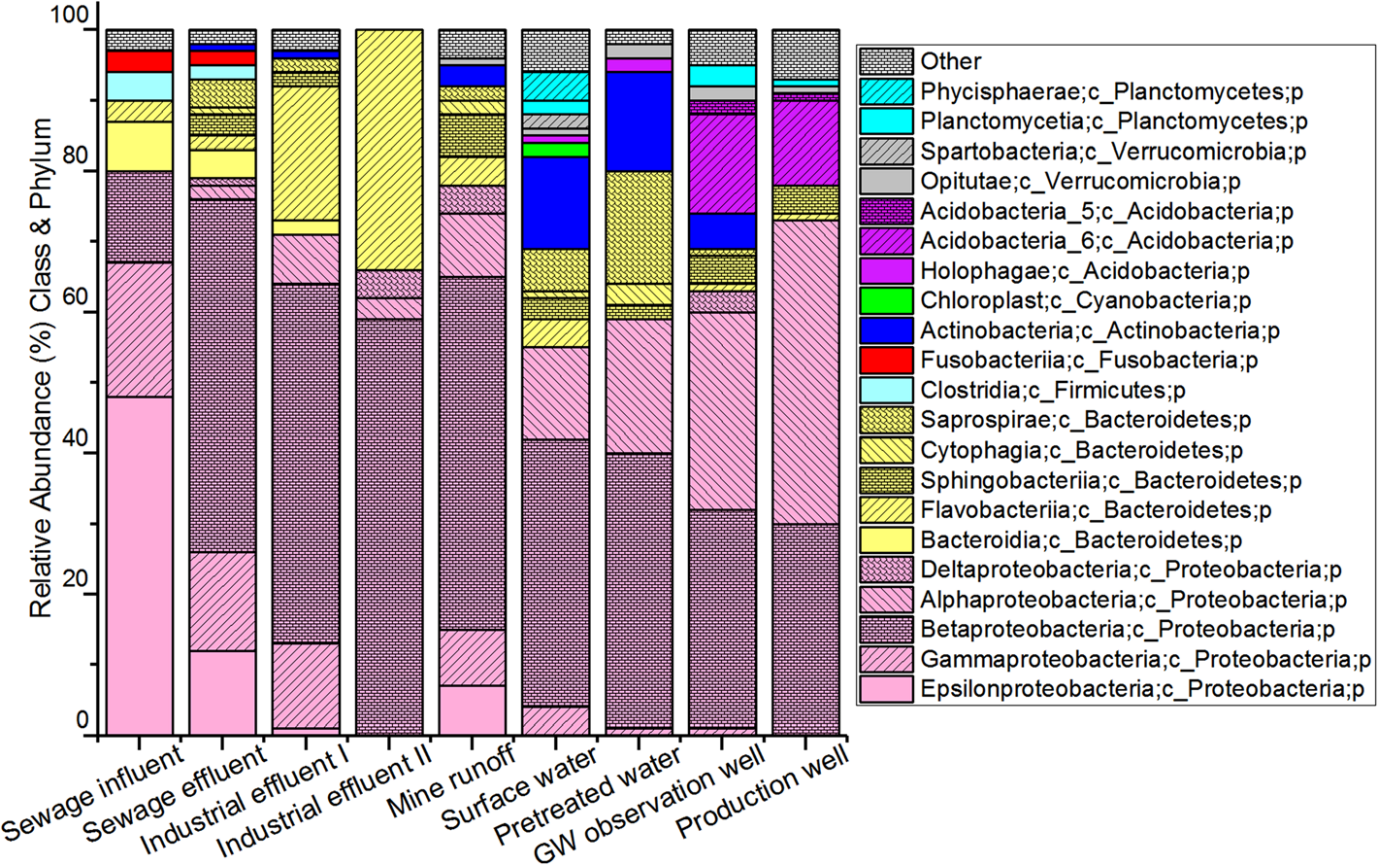
Bacterial taxonomic structure at the class and phylum level in the sample groups. Others: Classes having read contribution less than 2% in all the samples

The bacterial phylum *Proteobacteria* was omnipresent, but the relative abundance of this phylum was significantly higher in sewage samples (influent and effluent: 79 and 80%), industrial effluent (I and II: 66 and 71%), mine runoff (78%), GW observation well (63%), and GW production well (73%) compared to surface water (55%) samples (Fig. 2; *p* < 0.001, Kruskal-Wallis). The read proportion of *Proteobacteria* gradually increased through the DW production process. As demonstrated in Table 2, the seasonal effect on bacterial communities was significant only in surface water (R = 0.38, p < 0.001, ANOSIM). The seasonal variation of bacterial communities in surface water at the phylum and class levels is shown in Figure S7. The relative abundance of the *Proteobacteria* phylum was highest in the spring samples (64%) and lowest in the summer samples (45%). The difference was most visible in the relative abundance of the *Betaproteobacteria* class—the highest share in spring (49%) and the lowest in summer (28%).

Overall, within the *Proteobacteria* phylum, there was a large variation in bacterial classes in different sample groups (Fig. 2). *Epsilonproteobacteria* and *Gammaproteobacteria* were more abundant in sewage influent (48 and 19%, respectively) and effluent (12 and 14%, respectively) samples than in surface water and DW production process (Fig. 2; *p* < 0.001, Kruskal-Wallis). The abundance of *Epsilonproteobacteria* was less than 1% in the rest of the sample groups, except for mine runoff (7%). Furthermore, in most sample groups, the abundance of *Gammaproteobacteria* was less than 2%, except in the effluent from Industry I, mine runoff, and surface water, where their abundance was 12, 8, and 4%, respectively. *Betaproteobacteria* was detected in all sample groups, with the highest abundance in Industrial Effluent II (59%). The read proportion of class *Alphaproteobacteria* increased gradually as the drinking water treatment process proceeded (Fig. 2). The *Alphaproteobacteria* reads were more abundant in the AGR process samples (GW observation well: 28%, production well: 43%) than in the other groups (sewage: < 1%, industrial effluents: 2–7%, and mine runoff: 9%; surface water: 13%, pretreated: 19%) (Fig. 2; *p* < 0.001, Kruskal-Wallis).

**Fig. 3:**
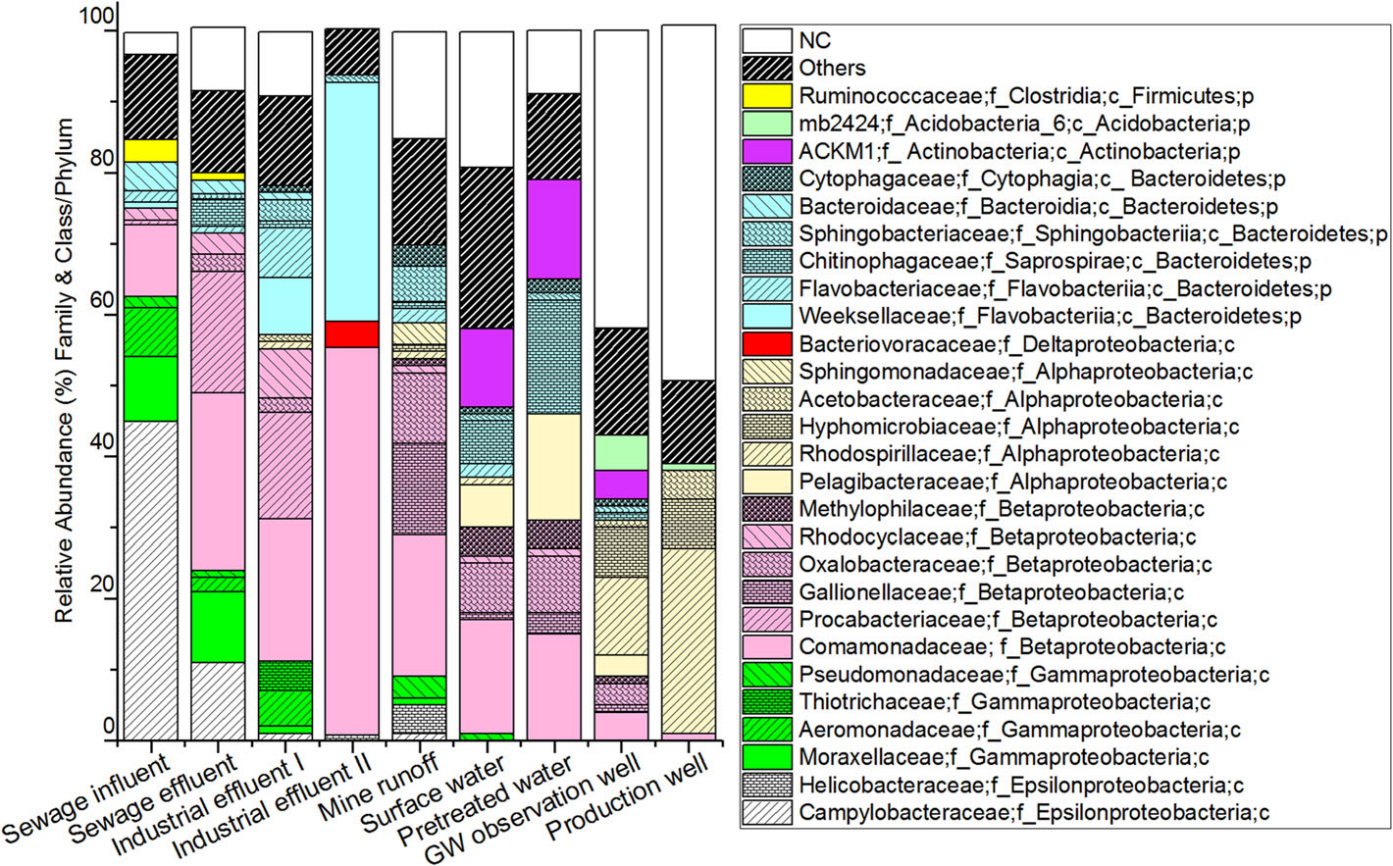
Bacterial taxonomic structure at the family level in the sample groups. Others: Families having a read contribution of less than 2% in all the samples. NA: Not assigned

The taxonomic classification was not possible for all reads at the family level, although the reads were assigned to the phyla and class levels (Fig. 3). The families *Campylobacteraceae* (influent: 45%, effluent: 11%, other sample groups: < 2%) and *Moracellaceae* (sewage: ~ 10%, other sample groups: < 1%) were mostly detected in the sewage samples. Among the *Betaproteobacteria*, the *Comamonadaceae* family was detected in all sample groups (Fig. 3), although it was more abundant in effluent and surface water than in samples from the DW production process (Fig. 3). Furthermore, the relative abundance of *Pelagibacteraceae* (*Alphaproteobacteria*) was 6% in surface water, 15% in pretreated water, and 3% in GW observation well samples. The relative abundance of members of the *Rhodospirillaceae* family (*Alphaproteobacteria*) was high in the production well (26%) and the GW observation well (11%) samples.

Among the studied samples, *Bacteroidetes* was the second-most abundant phylum (Fig. 2). Their reads were more abundant in the industrial samples (I: 25%, II: 34%) than in the surface water samples (14%). Their proportion increased from surface water to pretreated water (21%) but gradually decreased in the GW observation wells (6%) and production well (5%) samples. Within the *Bacteroidetes* phylum, some relative abundance patterns were noted at the class level: *Flavobacteriia* was solely detected in industrial effluent (I: 19%, II: 34%), while *Bacteroidia* was common in sewage (influent: 7%, effluent: 4%); *Saprospirae* was detected in surface water (6%) and pretreated water (16%). At the family level (Fig. 3), *Weeksellaceae* (*Flavobacteriia*) was dominant in the industrial effluent (I: 8% and II: 34%).

*Actinobacteria* was the third-most dominant phylum (Fig. 2), and most sequences belong to the *ACKM1* family (*Actinobacteria* class). The relative abundance of members of this phylum was higher in surface water (13%) and pretreated water (14%) than in the rest of the samples (≤5%). Of the other phyla, *Acidobacteria* was significantly more abundant in the GW observation well (16%) and production well (13%) samples than in other samples where the relative abundance of *Acidobacteria* was ≤2% (Fig. 2). Other less represented phyla were *Planctomycetes* (6%), *Verrucomicrobia* (3%), and *Cyanobacteria* (2%) in surface water and *Fusobacteria* and *Firmicutes*, which were solely detected in municipal sewage samples with a relative abundance of ≤4%.

### Core bacterial communities

**Fig. 4:**
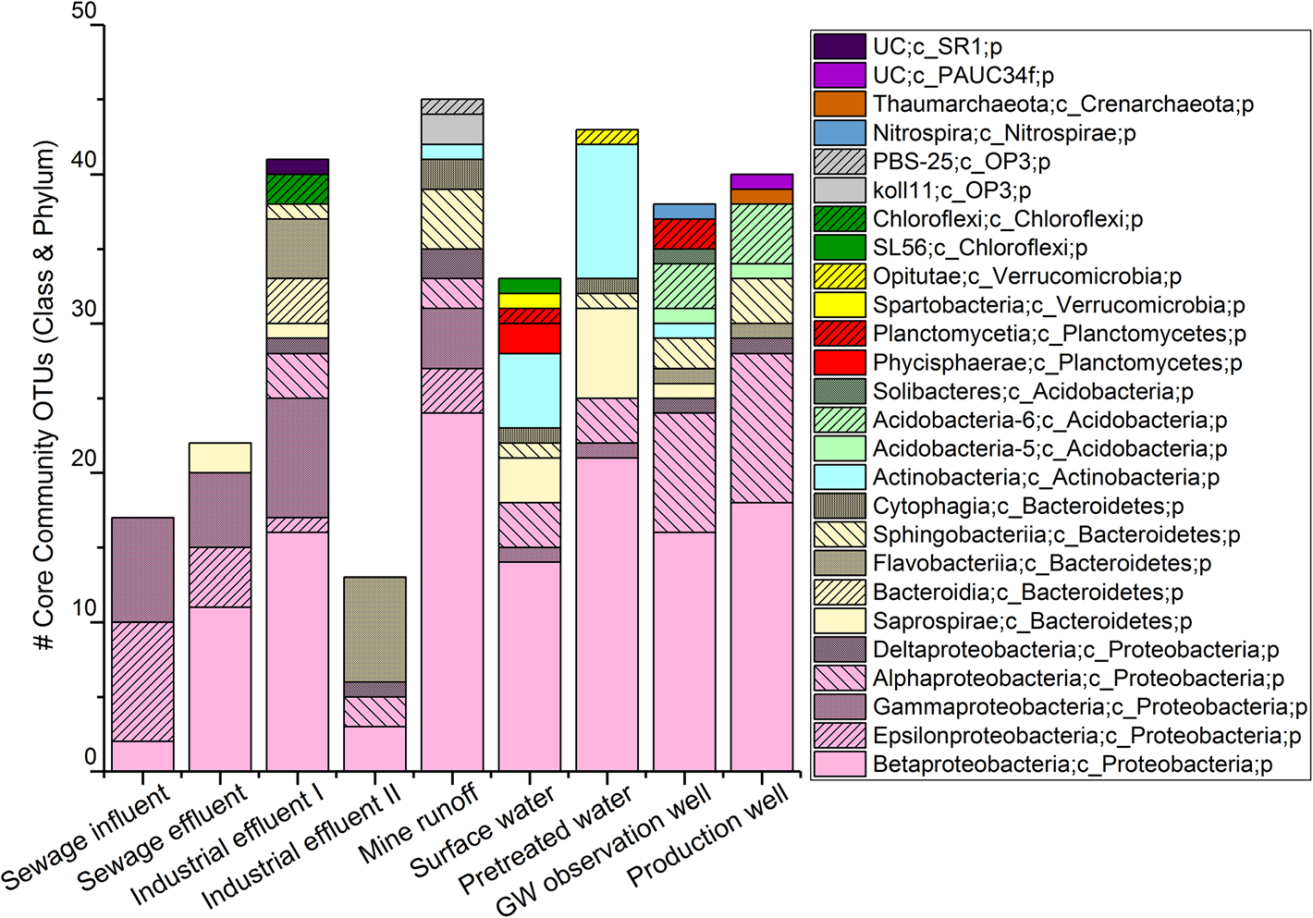
The number of core communities OTUs classified into class and phylum level

Core bacterial communities are reported here as the number of core OTUs at the class level (Fig. 4), while the OTUs having the highest sample prevalence or highest detection frequency in the sample group are reported at the family level (Table S6). Further details for different taxa levels are presented in supplementary data sheets S1, S2, S3, S4, S5, S6, S7, S8, S9 and S10.

At the class level, *Betaproteobacteria* was identified as a core member in all sample groups (Fig. 4). In the sewage influent samples, eight out of the 17 total core OTUs belonged to the class *Epsilonproteobacteria,* seven belonged to *Gammaproteobacteria,* and two belonged to *Betaproteobacteria* (Fig. 4, Supplemental Data Sheet S1). Regarding detection frequency, OTUs belonging to the *Campylobacteraceae* and *Aeromonadaceae* families were detected in all sewage influent samples (Table S6). In the sewage effluent samples of the 22 total core OTUs, *Betaproteobacteria* had ten*, Gammaproteobacteria* had five, and *Epsilonproteobacteria* had four core OTUs (Fig. 4, Supplemental Data Sheet S2). Based on the highest detection frequency in the sewage effluent, OTUs belonging to families *Campylobacteraceae* (*Epsilonproteobacteria* class, 83%), *Comamonadaceae* (*Betaproteobacteria* class, 73%), and *Moraxellaceae* (*Gammaproteobacteria* class, 73%) were the most prevalent (Table S6).

From surface water, fourteen *Betaproteobacteria* and five *Actinobacteria* core OTUs had the highest prevalence among the total number of core OTUs (*n* = 33; Fig. 4 and Supplemental Data Sheet S6). OTUs from families *Methylophilaceae* (98%), *Pelagibacteraceae* (96%), and *Comamonadaceae* (96%) had the highest detection frequency in surface water samples (Table S6). Among the pretreated water, *Betaproteobacteria* (21), *Actinobacteria* (9), and *Saprospirae* (6) had the highest number of core OTUs (*n* = 43, Fig. 4 and Supplemental Data Sheet S7). Core OTUs from families *Chitinophagaceae*, *Oxalobacteraceae*, *Pelagibacteraceae,* and *Methylophilaceae* were detected in all pretreated samples (Table S6). Pretreated water and surface water had some common core OTUs from families *ACK-M1*, *Methylophilaceae*, *Comamonadaceae,* and *Pelagibacteraceae* (supplemental data sheets S6 and S7).

In the GW observation well samples, classes *Betaproteobacteria* (16) and *Alphaproteobacteria* (8) and phyla *Acidobacteria* (6) and *Bacteroidetes* (4) had the highest core OTU numbers out of a total of 38 core OTUs (Fig. 4)*.* OTUs from families *mb2424* (76%), *Rhodospirillaceae* (71%), and an unidentified family OTU from the *Betaproteobacteria* class (76%) had the highest detection frequency (Table S6). From production well samples, classes *Betaproteobacteria* (19) and *Alphaproteobacteria* (10) and phyla *Acidobacteria* (6) and *Bacteroidetes* (4) had the highest core OTU numbers out of a total of 40 core OTUs (Fig. 4)*.* OTUs from families *Rhodospirillaceae*, *Acetobacteraceae,* and an unidentified family OTU from the *Betaproteobacteria* class had the highest detection frequency and were detected from all samples of this group (Table S6). More than half of the core OTUs from the GW observation well and production well samples were in common (supplemental data sheets S8, S9, and S10). From these samples, the majority of the core OTUs belonging to the *Betaproteobacteria* class were not identified at the lower taxonomic levels.

### Predicting functional diversity

**Fig. 5:**
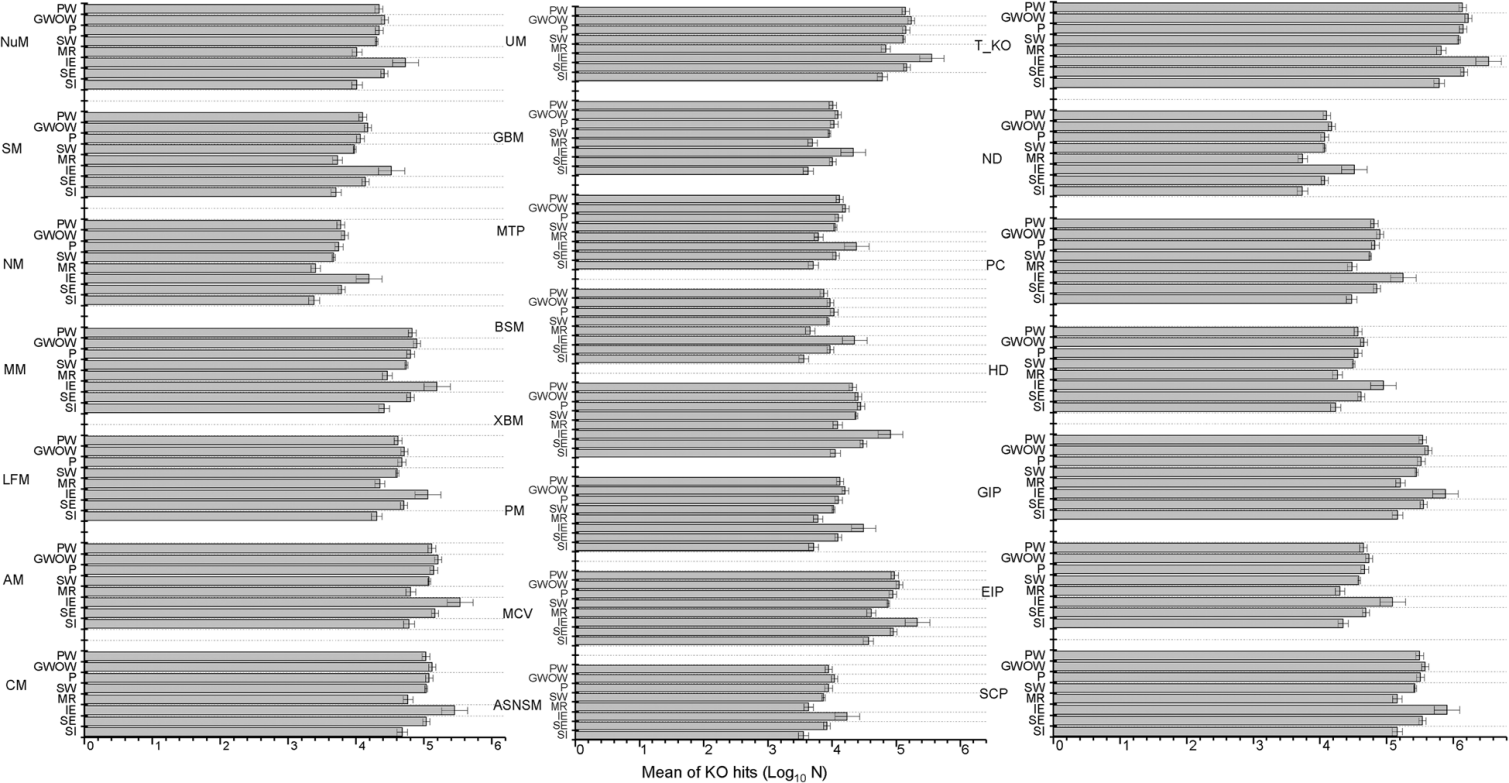
Mean KO hits of each function category in different sample categories. NuM = Nucleotide metabolism, SM = Sulfur metabolism, NM = Nitrogen metabolism, MM = Methane metabolism, LFM = Lipid and Fatty acid metabolism, AM = Amino acid metabolism, CM = Carbohydrate Metabolism, UM = Unclassified metabolism, GBM = Glycan Biosynthesis and Metabolism, MTP = Metabolism of Terpenoides and Polyketides, BSM = Biosynthesis of Secondary Metabolites, XBM = Xenobiotics Metabolism, PM = Pyrimidine Metabolism, MCV = Metabolism of cofactors and Vitamins, ASNSM = Amino Sugar and Nucleotide Sugar Metabolism, T_KO = Total KO hits, ND = new to dataset, PC = poorly characterized, HD = Human disease related, GIP = Genetic Information processing, EIP = Environmental Information processing, SCP = Signaling and cellular process. In the sample category, SI = Sewage Influent, SE = Sewage Effluent, IE = Industrial Effluent, ME = Mine Effluent, SW = Surface Water, P = Pretreated, PDT = Production Tube, PDW = Production Well. Error bar is calculated as σ/√n

A total of 6885 KEGG orthologs were obtained from the PICRUSt analysis. Among them, 1741 orthologs had zero KO hits in all samples. Furthermore, 3344 KEGG orthologs (99% KO hits out of a total of 6885) had KO hits of 500 or more in at least one sample out of the 230 studied samples. The orthologs were identified and grouped into three subgroups based on different KEGG functional gene ontology affiliations (i.e., metabolism, cellular process, and environmental process) and further divided into 21 different functional categories in each sample group (Fig. 5) and each season (Table S7). All predictive functional categories identified were higher in industrial effluents than in the rest of the sample groups. The categories of predictive functions did not differ significantly between surface water, pretreated water, GW observation well, and production well sample categories (Fig. 5). When all samples were considered (*n* = 230), KO hits for most of the predicted functions were significantly lower in the spring samples than in the other seasons, while the KO hits were not significantly different among these three seasons (Table S7).

### Detection of potential health-related bacteria - PHRB

**Fig. 6:**
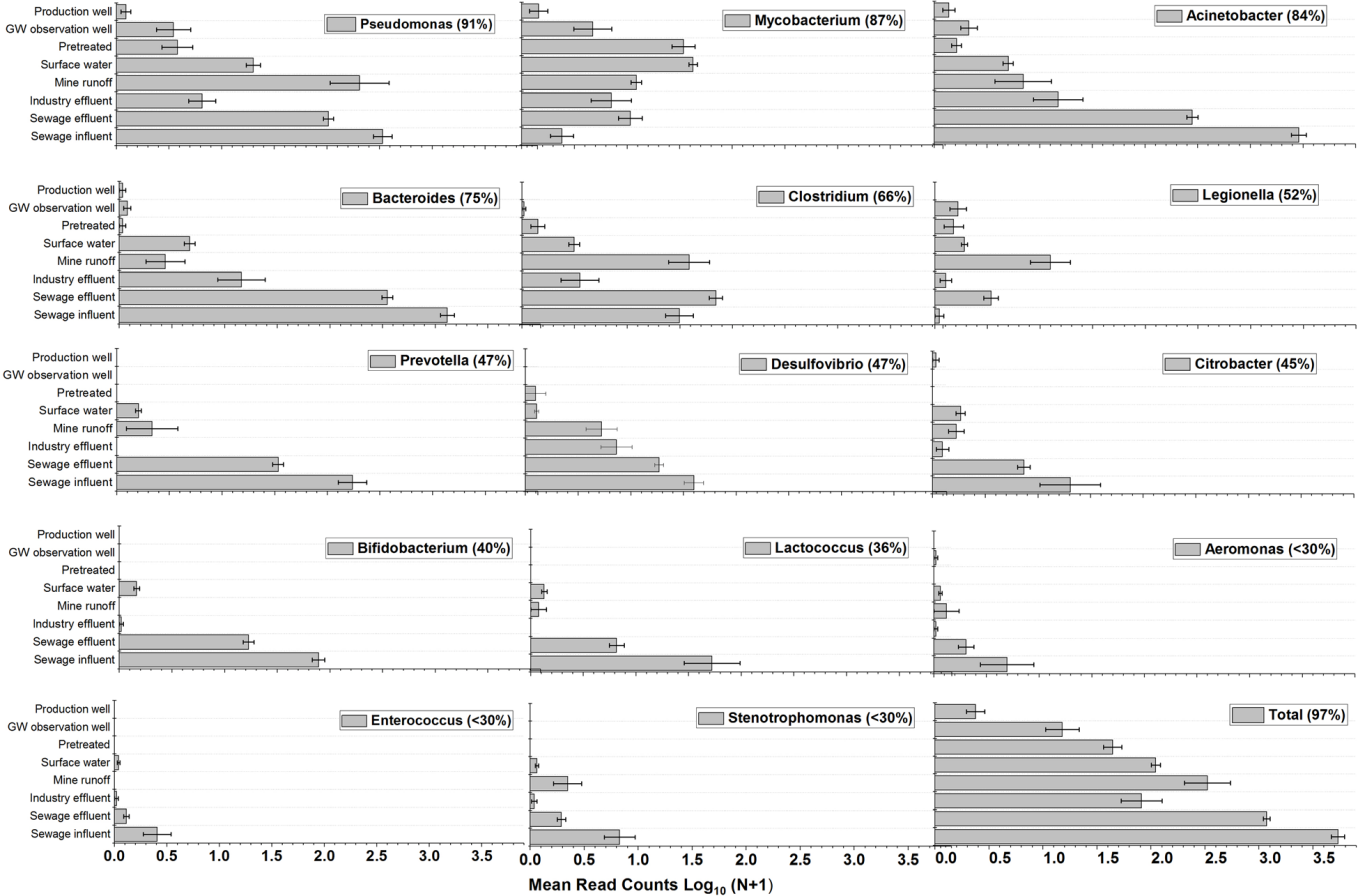
The mean read counts (with standard error) of PHRB in sample groups. The percentage shows the detection frequency within the whole dataset (number of samples, *n* = 230). See Supplementary Table S3 for further information about the potential health-related bacteria (PHRB)

Among a total of 42 PHRB genera screened, 20 were detected (Supplemental Table S3). The variation of PHRB in different sample groups is shown in Fig. 6. Within all samples (*n* = 230), a total of 145,116 bacterial reads (2.4% of the total bacterial reads) were determined to belong to PHRB. *Pseudomonas* spp., *Mycobacterium* spp., and *Acinetobacter* spp. were the most frequently detected PHRB genera and were detected in 210, 200, and 193 samples, respectively. When the read counts were considered, *Acinetobacter* spp., *Bacteroides* spp., and *Pseudomonas* spp. had the three highest read counts at 470370, 37765, and 25,540, respectively. *Acinetobacter*, *Bacteroides,* and *Pseudomonas* also had the top three in OTU numbers with 47, 45, and 57 OTUs, respectively, out of a total of 221 OTUs belonging to PHRB detected in this study. *Arcobacter* spp. was among the most prevalent groups in sewage influent and sewage effluent samples. Most of the *Campylobacteraceae* family reads from these groups of samples belonged to *Arcobacter* spp. (Fig. 3). Furthermore, *Arcobacter* spp. was among the most commonly found core OTUs from municipal sewage samples (supplemental data sheets S1 and S2).

The numbers of all detected PHRB reads were significantly different between the sample groups (*p* < 0.001, Kruskal-Wallis). All detected PHRB genera except *Legionella* spp. and *Mycobacterium* spp. had higher read counts in the sewage influent samples compared to the other sample groups (Fig. 6). The highest read counts of *Legionella* spp. and *Mycobacterium* spp. were detected in the mine runoff and surface water samples, respectively. *Clostridium* spp. reads were more abundant in sewage effluent, sewage influent, and mine runoff samples than in the rest of the samples. Overall, the number of PHRB reads was lower in the production well samples than in the other samples. Most of the PHRB genera had a clear seasonal pattern (Table S8). The median read counts of most PHRB genera were lower in the summer samples than in the other seasons.

## Discussion

This study demonstrated the distinct bacterial diversity, taxonomic structure, predicted enzymatic function, and PHRB in different water sample types (e.g., mainly sewage, industrial effluents, surface water, mine runoff, and AGR-based drinking water). These findings are in accordance with earlier studies [26, 30, 55], where distinct bacterial communities found in groundwater, surface water, treated effluent, treated drinking water, mine runoff, and household tap water.

Ecological conditions affect bacterial diversity and community structure [55]. For example, surface water experiences large changes in temperature and daylight hours in different seasons of the year. However, such changes did not affect the other water types in the present study as the surface water was the only sample group where seasonal changes affected the bacterial composition. In contrast, groundwater (i.e., observation and production wells sampled in this study) may have more uniform physico-chemical conditions—such as anoxic, dark, and oligotrophic—with a constant temperature. Furthermore, the sewage samples and industrial effluents have a lower dissolved oxygen concentration due to high biological and chemical oxygen demand. Also, the controlled and uniform environment may shape the bacterial communities. For example, the chemical toxicity due to the use of biocides may explain the low taxonomic diversity in industrial samples [24]. In such cases, bacterial groups having a wide metabolic capacity may survive, such as members of the *Comamonadaceae* and *Rhodocyclaceae* families (*Betaproteobacteria* class) detected in the industrial effluent samples (Fig. 3). In future studies, coupling the nutrient analysis results to the fecal microbial analysis could provide additional information about the surface water contamination events [6].

Surface water samples had a high taxonomic diversity, as determined by the Shannon diversity index (Table 3). The higher taxonomic diversity in surface water than in the drinking water treatment process samples was consistent with earlier findings [17, 30]. In addition to higher alpha-diversity in raw water than in treated drinking water (Table 3), Gülay et al., [17] reported the negative relationship between bacterial diversity and energy input. Thus, the noted lowest alpha-diversity of bacteria in GW observation and production wells in our study is expected, as the available energy sources for bacterial cells decrease consistently during the water purification process. The low alpha-diversity in the drinking water samples may imply biologically stable water [45]. Traditionally, the biological stability is mainly regulated by monitoring the heterotrophic plate count (HPC [51];), the parameter being able to detect only culturable micro-organisms [45]. The use of alpha-diversity may provide a better idea of the biological stability of water than HPC as high-throughput sequencing measures both culturable and difficult to culture autotrophic and heterotrophic bacteria [45].

The lower taxonomic diversity of surface water in the summer season (Table S5) and seasonal variation in the community structure have previously been reported [35, 54]. The lower alpha-diversity in the summer season can be due to bacterial predation [33]. The Finnish river ecosystem can become more active during summer and may increase the eukaryotes that graze on bacteria, which can control bacterial diversity. Another explanation can be inactivation due to higher solar radiation and longer daylight exposure.

### Characteristics of the detected bacterial community members

Bacterial communities belonging to classes *Epsilonproteobacteria*, *Gammaproteobacteria*, *Fusobacteriia*, *Bacilli*, *Clostridia*, and *Bacteroidia* were mostly detected from municipal sewage samples and were reduced during the sewage treatment process (Fig. 2). These bacterial classes have also been detected previously in raw and treated sewage [33, 48]. The significant reduction of these bacterial groups during the sewage treatment process is not surprising. Many of these bacteria prefer to grow in the anaerobic gut environment, while wastewater treatment with activated sludge is an aerobic process. The detection of these bacterial groups in mine runoff may indicate the poor management of sanitary waste in the mine area (Fig. 2).

Most *Epsilonproteobacteria* detected in sewage samples are related to the genus *Arcobacter*, many of which may be considered commensal, pathogenic, or free-living. These bacteria can grow in micro-aerobic, anaerobic, or aerobic conditions. Their optimum temperature ranges from 25 to 42 °C. The majority of the *Gammaproteobacteria* reads of sewage effluent samples were from the families *Aeromonadaceae* and *Moraxellaceae*. *Aeromonadaceae* was also abundant in industrial samples (Fig. 3). Members of these two groups can be pathogenic, commensal, or free-living [25, 47]. Some *Aeromonadaceae* are strict aerobes, while others are facultative anaerobes. This group can be mesophilic or psychrophilic [25].

Members of the *Comamonadaceae* family (*Betaproteobacteria* class) were detected in all sample groups but were most abundant in industrial effluents, municipal effluent, and mine runoff (Fig. 3). Many are free-living and exhibit wide metabolic capabilities such as aerobic organotroph, anaerobic denitrifier, iron reducer, hydrogen oxidizer, photoautotroph, photo-heterotroph, or fermenter [50]. Industrial and sewage effluent also had reads of the *Rhodocyclaceae* family (*Betaproteobacteria* class), which are photo-heterotrophs, plant-associated, nitrogen-fixing aerobes capable of utilizing varying sources of organic carbon and energy [41]. Members of the *Weeksellaceae* family (*Bacteroidetes* phylum), known to be aerobic and free-living environmental bacteria [42], were present in industrial effluent samples (Fig. 3). Earlier studies reported this group from sewage and activated sludge [3, 42]. Wang et al. [48] suspected the higher organic carbon may favor the growth of the *Flavobacteriia* class in aquatic systems.

The detection of the bacterial phyla *Proteobacteria*, *Actinobacteria,* and *Bacteroidetes* in surface water (Fig. 2) was consistent with earlier findings [26, 48, 54]. In contrast, Abia et al. [1] reported a higher relative abundance of *Alphaproteobacteria* than *Betaproteobacteria* in surface water samples. Additionally, they reported relatively higher (70%) *Proteobacteria* reads of river water samples than in our study. The relatively lower proportion of *Proteobacteria* phylum in surface water and pretreated samples than in other sample groups can be due to the presence of environmental bacteria like *Actinobacteria*. The *Actinobacteria* phylum was the most abundant among the Gram-positive bacteria in surface water. The identified reads were mostly heterotrophic or symbiotic with plants such as nitrogen-fixing bacteria belonging to the family *ACKM1* [18]. Although an attempt was made to filter out the chloroplast reads, it was detected in surface water samples (Fig. 2). The *Chloroplast* sequences are closely related to *Cyanobacteria*; they can originate from cyanos and are therefore difficult to remove from the data completely. There could be classification problems in the taxonomic databases related to these 16S rRNA sequences. *Chloroplast* reads have also been reported in earlier 16S rRNA gene amplicon sequencing studies [56].

Other major groups found in surface water were the families *Oxalobacteraceae* and *Methylophilaceae* (*Betaproteobacteria* class). These are free-living environmental bacteria groups known for their wide range of phenotypic properties and include aerobic or micro-aerobic, facultative anaerobic, heterotrophic, and mesophilic members [4, 14]. The *Methylophilaceae* can utilize methanol or methylamine as a source of carbon and energy and have been reported in numerous environments including activated sludge [14]. The close similarity in bacterial communities in surface water samples with pretreated samples implies that the pretreatment process does not effectively change the bacterial communities. The seasonal variation in the taxonomic profile in surface water was consistent with an earlier study [54].

The relatively high proportion of the *Proteobacteria* phylum (73%) in the AGR production well samples was higher than the 47% reported in Ma et al. [36] from samples originating from drinking water production with river water with a traditional treatment process. The gradual increase in *Proteobacteria* reads from surface water, pretreated water, GW observation well, and (finally) the production well (Fig. 3) suggests that other bacterial groups may not tolerate the change in the environment. The majority of the bacterial reads belonged to *Betaproteobacteria* classes from groundwater samples (observation and production wells) that were not identified at deeper taxonomic levels than class and order (Fig. 3). *Rhodospirillaceae* (within the *Alphaproteobacteria* class) was one of the most abundant families in groundwater. This group is anaerobic chemoheterotrophic under dark conditions and heterotrophic under aerobic conditions [40]. Members of the metabolically diverse soil bacterial groups *Acidobacteria*-5 and *Acidobacteria*-6 were also found in groundwater samples (Fig. 2), which were also previously observed by Kielak et al. [31].

### The distribution of bacteria with public health relevance in the samples

The use of the 16S rRNA gene-based high-throughput method offers the possibility to simultaneously get information from multiple PHRB groups. However, in general, a much larger volume of water is analyzed when enumerating pathogens from environmental samples. Therefore, the resolution of the taxonomic assignment of the 16S rRNA gene sequence might not be high enough to reliably identify and quantify the pathogens [23]. In the present study, the abundance of PHRB read counts in sewage samples illustrates municipal sewage as a source of PHRB. The abundance of *Mycobacterium* and *Legionella* reads in environmental samples was not surprising as these two genera are independent of fecal contamination. Although the high-throughput sequencing method used here gives information only up to the genus level, the detection of genera that house some pathogens can raise the suspicion that the ecological conditions may also be favorable for some of the pathogenic species to survive. In addition, the bacterial diversity in drinking water can also have some human health benefits. For example, Hertzen et al. [20] claimed that bacterial diversity in drinking water may reduce atopy among the public. Furthermore, the PHRB listed in this study necessarily does not have to have any negative public health impacts. Of the included genera, *Citrobacter, Klebsiella, Escherichia,* and *Enterobacter* are better known as a fecal indicator bacteria of water quality and not considered pathogens. Additionally, genera like *Bifidobacterium* and *Lactococcus* are gut commensal communities with limited pathogenicity [23].

### Variation in predicated functions

The highest mean KO values of all types of predicted functions were detected in the industrial effluent samples. Indeed, in industrial water systems, microbes might need to activate more pathways to enable their survival in that environment [11]. For example, activation of terpene and secondary metabolite pathways was observed. This suggests a selection towards bacterial groups capable of degrading some toxic compounds that may be present in industrial waste. This selection possibly explains the relatively lower diversity recorded in the industrial effluents. Additionally, other toxic components of industrial waste may promote the production of the secondary metabolites (such as antibiotics and bacteriocins) used by the producing bacteria to eliminate competitors [21]. The other predicted functions, such as those that relate to nitrogen, sulfur, amino acid, and lipid metabolisms, were relatively higher in the industrial effluents, suggesting that some of the enriched bacterial groups are engaged in the utilization of many available carbon and energy sources needed for growth. In contrast, in our data, sewage influent and mine runoff samples dramatically reduced these predicted functions, suggesting that the bacterial communities in these samples are facing greater environmental perturbations. While these communities formed different clusters, the predicted functions and taxonomic groups observed suggest that these are relatively complex communities capable of withstanding seasonal changes, in part due to the diversity of functional redundancies.

Furthermore, surprisingly, we did not notice that the predictive ecological functions of bacterial communities were independent of the sample groups and taxonomic variation. Although our study did not determine the accuracy of the prediction, the developers of the PICRUSt tool [34] claimed about 80% accuracy of the truth for the prediction. These results imply that a wide bacterial community range in the aquatic ecosystem performs similar ecological functions, and taxonomic variation may have a low effect on overall ecological functions. Furthermore, the poorly classified OTUs at the genus level—38% overall; lowest in groundwater (10%) and highest in mine runoff (60%)—may have an effect on similar KO hits on various types of sample groups. PICRUSt can help to predict the presence of genetic functions in difficult-to-classify lower taxonomic groups, even if representative full genomes have not been completely characterized by using gene sequences found in closely related bacterial groups. The reason behind the lower predicted function during the spring season is unknown.

## Conclusions

- This study described the seasonal composition of bacterial communities, their predicted functional profiles, and the presence of PHRB in samples collected within the Kokemäenjoki River watershed impacted by diverse anthropogenic pollutant sources.
- The reduction in PHRB reads and alpha-diversity indices in the process from raw water to DW production implies the efficiency of AGR as a drinking water treatment process.
- In surface water samples, the seasonal variation in bacterial diversity was significant, and the share of PHRB reads was lower in summer than in other seasons.
- Effective sewage treatment is a necessity for protecting surface water quality.

## Supplementary Information

**Additional file 1: Table S1.** Geographical locations of sampling area. **Table S2.** Climatic condition at nearby stations. **Table S3.** Public health-related bacteria (PHRB) and their detection frequencies. **Table S4.** Correlation between various alpha diversity indices. **Table S5.** Seasonal variation of sequencing reads and alpha diversity indices in surface water. **Table S6.** Top 3 highest sample prevalence OTUs in each sample group. **Table S7.** Seasonal variation of KO hits. **Table S8.** Seasonal variation of PHRB read counts in surface water. **Figure S1.** Rarefaction curve of 16S rRNA gene amplicon sequences in sample groups. **Figure S2.** Variation in read counts, Chao1, Shannon index, and Simpson index in sample groups. **Figure S3.** Variation in OTUs number, Observed index, and ACE index in sample groups. **Figure S4.** Seasonal variation of read counts, Chao1, Shannon index, and Simpson index in surface water. **Figure S5.** Seasonal variation of OTUs number, Observed index, and ACE index in surface water. **Figure S6.** Seasonal variation of bacterial taxa in surface water. **Figure S7.** Bacterial diversities in different surface water types.

**Additional file 2: Supplemental Data Sheet: Core bacterial communities. Supplemental Data Sheet S1.** Municipal influent. **Supplemental Data Sheet S2.** Municipal effluent. **Supplemental Data Sheet S3.** Industrial effluent I. **Supplemental Data Sheet S4.** Industrial effluent II. **Supplemental Data Sheet S5.** Mine runoff. **Supplemental Data Sheet S6.** Surface water. **Supplemental Data Sheet S7.** Pretreated water. **Supplemental Data Sheet S8.** Groundwater (combine groundwater observation and production wells). **Supplemental Data Sheet S9.** Groundwater observation wells. **Supplemental Data Sheet S10.** Groundwater production wells.

## Data Availability

The raw reads generated during this study are available in the Short Read Archive (SRA) of NCBI under BioSample accession numbers from SAMN14132458 to SAMN14132699 under the BioProject PRJNA607422.
