# Supplementary material for: Bacterial diversity and predicted enzymatic function in a multipurpose surface water system – from wastewater effluent discharges to drinking water production
Source: Environ Microbiome. 2021 May 22;16:11. doi: 10.1186/s40793-021-00379-w (PMC8140503; doi:10.1186/s40793-021-00379-w)
Supplement: Supplementary file 1 — Additional file 1: Table S1. Geographical locations of sampling area. Table S2. Climatic condition at nearby stations. Table S3. Public health-related bacteria (PHRB) and their detection frequencies. Table S4. Correlation between various alpha diversity indices. Table S5. Seasonal variation of sequencing reads and alpha diversity indices in surface water. Table S6. Top 3 highest sample prevalence OTUs in each sample group. Table S7. Seasonal variation of KO hits. Table S8. Seasonal variation of PHRB read counts in surface water. Figure S1. Rarefaction curve of 16S rRNA gene amplicon sequences in sample groups. Figure S2. Variation in read counts, Chao1, Shannon index, and Simpson index in sample groups. Figure S3. Variation in OTUs number, Observed index, and ACE index in sample groups. Figure S4. Seasonal variation of read counts, Chao1, Shannon index, and Simpson index in surface water. Figure S5. Seasonal variation of OTUs number, Observed index, and ACE index in surface water. Figure S6. Seasonal variation of bacterial taxa in surface water. Figure S7. Bacterial diversities in different surface water types. [file 40793_2021_379_MOESM1_ESM.docx]

Supplementary Material

**Bacterial diversity and predicted enzymatic function in a multipurpose surface water system – from wastewater effluent discharges to drinking water production**

Ananda Tiwari, Anna-Maria Hokajärvi, Jorge Santo Domingo, Michael Elk, Balamuralikrishna Jayaprakash, Hodon Ryu, Sallamaari Siponen, Asko Vepsäläinen, Ari Kauppinen, Osmo Puurunen, Aki Artimo, Noora Perkola, Timo Huttula, Ilkka T. Miettinen and Tarja Pitkänen

Table S1. Latitude and longitude of surface water sampling locations.

| No | Sampling site | Latitude | Longitude |
| --- | --- | --- | --- |
| 1 | Ratinanvuolle | 6823179 | 327455 |
| 2 | Pyhäjärvi | 6821482 | 325614 |
| 3 | Rajasaari | 6820277 | 318752 |
| 4 | Sotkanvirta | 6817114 | 312630 |
| 5 | Nokiankoski (upstream) | 6820104 | 314890 |
| 6 | Nokiankoski (downstream) | 6819577 | 310206 |
| 7 | Siuronkoski | 6821375 | 304678 |
| 8 | Hiedanvuolle | 6814187 | 290176 |
| 9 | Rautavesi | 6808403 | 284378 |
| 10 | Liekovesi | 6806482 | 277832 |
| 11 | Keikyä | 6800064 | 269326 |
| 12 | Karhiniemi | 6794212 | 267916 |
| 13 | Loimijoki | 6789165 | 268155 |
| 14 | Kojo, Kolsi | 6802112 | 251104 |

**Table S2.** The climatic conditions at the cities of Tampere and Turku are located around the study area, south-western part of Finland.

| **Season** |  | **Daylight^1^**  **(hours: minutes)** | | **Temperature^2^ (°C)** | | **Precipitation^2^ (mm)** | |
| --- | --- | --- | --- | --- | --- | --- | --- |
|  |  | Tampere | Turku | Tampere | Turku | Tampere | Turku |
| Autumn | September | 12:56 | 12:54 | 10.5 | 11.2 | 59.8 | 62.8 |
|  | October | 10:04 | 10:09 | 5.3 | 6.4 | 66.1 | 75.4 |
|  | November | 7:13 | 7:27 | -2.0 | 1.3 | 53.6 | 72.9 |
| Winter | December | 5:25 | 5:47 | -4.1 | -2.1 | 46.1 | 64.9 |
|  | January | 6:20 | 6:37 | -6.1 | -4.0 | 45.3 | 54.4 |
|  | February | 9:02 | 9:10 | -6.7 | -4.8 | 30.8 | 37.6 |
| Spring | March | 11:45 | 11:45 | -2.4 | -1.4 | 33.5 | 38.7 |
|  | April | 14:45 | 14:38 | 3.5 | 4.0 | 31.4 | 31.0 |
|  | May | 17:33 | 17:16 | 9.9 | 10 | 39.7 | 37.6 |
| Summer | June | 19:26 | 18:59 | 14.4 | 14.4 | 64.3 | 55.4 |
|  | July | 18:33 | 18:12 | 17.4 | 17.5 | 74.6 | 77.6 |
|  | August | 15:53 | 15:43 | 15.6 | 16.2 | 74.7 | 78.2 |

^1^Daylight duration of 15^th^ of each month of 2013 (data source: <https://www.timeanddate.com/>). ^2^Monthly average temperature and precipitation between 1981-2010 (data source: <https://en.ilmatieteenlaitos.fi/home>).

**Table S3.** The selection of public health-related bacteria (PHRB) and their detection frequency within the whole data (n = 230). ND = not detected.

| **No.** | **Selected PHRB genus^1^** | **Detection Frequency %** | **Family** | **Order** | **Class** | **Phylum** |
| --- | --- | --- | --- | --- | --- | --- |
| 1 | *Mycobacterium* spp. | 87 | *Mycobacteriaceae* | *Actinomycetales* | *Actinobacteria* | *Actinobacteria* |
| 2 | *Bifidobacteria* spp. | 40 | [*Bifidobacteriaceae*](https://en.wikipedia.org/wiki/Bifidobacteriaceae) | [*Bifidobacteriales*](https://en.wikipedia.org/wiki/Bifidobacteriales) | [*Actinobacteria*](https://en.wikipedia.org/wiki/Actinobacteria) | [*Actinobacteria*](https://en.wikipedia.org/wiki/Actinobacteria) |
| 3 | *Actinomyces* spp. | ND | [*Actinomycetaceae*](https://en.wikipedia.org/wiki/Actinomycetaceae) | [*Actinomycetales*](https://en.wikipedia.org/wiki/Actinomycetales) | *Actinobacteria* | *Actinobacteria* |
| 4 | *Propionobacterium* spp. | ND | [*Propionibacteriaceae*](https://en.wikipedia.org/wiki/Propionibacteriaceae) | [*Propionibacteriales*](https://en.wikipedia.org/w/index.php?title=Propionibacteriales&action=edit&redlink=1) | *Actinobacteria* | *Actinobacteria* |
| 5 | *Raoultella* spp. | ND | [*Propionibacteriaceae*](https://en.wikipedia.org/wiki/Propionibacteriaceae) | [*Propionibacteriales*](https://en.wikipedia.org/w/index.php?title=Propionibacteriales&action=edit&redlink=1) | *Actinobacteria* | *Actinobacteria* |
| 6 | *Tsukamurella* spp. | ND | [*Tsukamurellaceae*](https://en.wikipedia.org/w/index.php?title=Tsukamurellaceae&action=edit&redlink=1) | *Actinomycetales* | *Actinobacteria* | *Actinobacteria* |
| 7 | *Bacteroides* spp. | 75 | *Bacteroidaceae* | *Bacteroidales* | *Bacteroidia* | *Bacteroidetes* |
| 8 | *Prevotella* spp. | 47 | *Prevotellaceae* | *Bacteroidales* | *Bacteroidia* | *Bacteroidetes* |
| 9 | *Clostridium* spp. | 66 | *Clostridiaceae* | *Clostridiales* | *Clostridia* | *Firmicutes* |
| 10 | *Lactococcus* spp. | 36 | [*Streptococcaceae*](https://en.wikipedia.org/wiki/Streptococcaceae) | [*Lactobacillales*](https://en.wikipedia.org/wiki/Lactobacillales) | [*Bacilli*](https://en.wikipedia.org/wiki/Bacilli) | *Firmicutes* |
| 11 | *Ruminococcus* spp. | 34 | [*Ruminococcaceae*](https://en.wikipedia.org/wiki/Ruminococcaceae) | *Clostridiales* | *Clostridia* | *Firmicutes* |
| 12 | *Lachnospiraceae* spp. | 32 | *Lachnospiraceae* | *Clostridiales* | *Clostridia* | *Firmicutes* |
| 13 | *Enterococcus* spp. | 14 | *Enterococcaceae* | [*Lactobacillales*](https://en.wikipedia.org/wiki/Lactobacillales) | [*Bacilli*](https://en.wikipedia.org/wiki/Bacilli) | *Firmicutes* |
| 14 | *Streptococcus* spp. | 5 | [*Streptococcaceae*](https://en.wikipedia.org/wiki/Streptococcaceae) | [*Lactobacillales*](https://en.wikipedia.org/wiki/Lactobacillales) | [*Bacilli*](https://en.wikipedia.org/wiki/Bacilli) | *Firmicutes* |
| 15 | *Lactobacillus* spp. | 3 | [*Lactobacillaceae*](https://en.wikipedia.org/wiki/Lactobacillaceae) | [*Lactobacillales*](https://en.wikipedia.org/wiki/Lactobacillales) | [*Bacilli*](https://en.wikipedia.org/wiki/Bacilli) | *Firmicutes* |
| 16 | *Eubacterium* spp. | ND | [*Eubacteriaceae*](https://en.wikipedia.org/wiki/Eubacteriaceae) | *Clostridiales* | *Clostridia* | *Firmicutes* |
| 17 | *Peptococcus* spp. | ND | [*Peptococcaceae*](https://en.wikipedia.org/wiki/Peptococcaceae) | *Clostridiales* | *Clostridia* | *Firmicutes* |
| 18 | *Peptostreptococcus* spp. | ND | [*Peptostreptococcaceae*](https://en.wikipedia.org/wiki/Peptostreptococcaceae) | *Clostridiales* | *Clostridia* | *Firmicutes* |
| 19 | *Staphylococcus* spp. | ND | [*Staphylococcaceae*](https://en.wikipedia.org/wiki/Staphylococcaceae) | [*Bacillales*](https://en.wikipedia.org/wiki/Bacillales) | [*Bacilli*](https://en.wikipedia.org/wiki/Bacilli) | *Firmicutes* |
| 20 | *Fusobacterium* spp. | 8 | [*Fusobacteriaceae*](https://en.wikipedia.org/wiki/Fusobacteriaceae) | [*Fusobacteriales*](https://en.wikipedia.org/wiki/Fusobacteria) | [*Fusobacteriia*](https://en.wikipedia.org/wiki/Fusobacteria) | [*Fusobacteria*](https://en.wikipedia.org/wiki/Fusobacteria) |
| 21 | *Pseudomonas* spp. | 91 | *Pseudomonadaceae* | *Pseudomonadales* | *Gammaproteobacteria* | *Proteobacteria* |
| 22 | *Acinetobacter* spp. | 84 | *Moraxellaceae* | *Pseudomonadales* | *Gammaproteobacteria* | *Proteobacteria* |
| 23 | *Legionella* spp. | 52 | *Legionellaceae* | *Legionellales* | *Gammaproteobacteria* | *Proteobacteria* |
| 24 | *Desulfovibrio* spp. | 46 | *Desulfovibrionales* | *Desulfovibrionales* | *Deltaproteobacteria* | *Proteobacteria* |
| 25 | *Citrobacter* spp | 45 | *Enterobacteriaceae* | *Enterobacterales* | *Gammaproteobacteria* | *Proteobacteria* |
| 26 | *Stenotrophomonas* spp. | 27 | [*Xanthomonadaceae*](https://en.wikipedia.org/wiki/Xanthomonadaceae) | [*Xanthomonadales*](https://en.wikipedia.org/wiki/Xanthomonadales) | *Gammaproteobacteria* | *Proteobacteria* |
| 27 | *Aeromonas* spp. | 17 | [*Aeromonadaceae*](https://en.wikipedia.org/wiki/Aeromonadaceae) | [*Aeromonadales*](https://en.wikipedia.org/wiki/Aeromonadales) | *Gammaproteobacteria* | *Proteobacteria* |
| 28 | *Vibrio* spp. | 2 | [*Vibrionaceae*](https://en.wikipedia.org/wiki/Vibrionaceae) | [*Vibrionales*](https://en.wikipedia.org/wiki/Vibrionaceae) | *Gammaproteobacteria* | *Proteobacteria* |
| 29 | *Aliivibrio* spp. | ND | [*Vibrionaceae*](https://en.wikipedia.org/wiki/Vibrionaceae) | [*Vibrionales*](https://en.wikipedia.org/wiki/Vibrionaceae) | *Gammaproteobacteria* | *Proteobacteria* |
| 30 | *Burkholderia* spp. | ND | [*Burkholderiaceae*](https://en.wikipedia.org/wiki/Burkholderiaceae) | [*Burkholderiales*](https://en.wikipedia.org/wiki/Burkholderiales) | [*Betaproteobacteria*](https://en.wikipedia.org/wiki/Betaproteobacteria) | *Proteobacteria* |
| 31 | *Campylobacter* spp. | ND | [*Campylobacteraceae*](https://en.wikipedia.org/wiki/Campylobacteraceae) | [*Campylobacterales*](https://en.wikipedia.org/wiki/Campylobacterales) | [*Epsilonproteobacteria*](https://en.wikipedia.org/wiki/Epsilonproteobacteria) | *Proteobacteria* |
| 32 | *Enterobacter* spp. | ND | *Enterobacteriaceae* | *Enterobacterales* | *Gammaproteobacteria* | *Proteobacteria* |
| 33 | *Escherichia* spp. | ND | *Enterobacteriaceae* | *Enterobacterales* | *Gammaproteobacteria* | *Proteobacteria* |
| 34 | *Francisella* spp. | ND | *Francisellaceae* | *Thiotrichales* | *Gammaproteobacteria* | *Proteobacteria* |
| 35 | *Grimontia* spp. | ND | [*Vibrionaceae*](https://en.wikipedia.org/wiki/Vibrionaceae) | [*Vibrionales*](https://en.wikipedia.org/wiki/Vibrionaceae) | *Gammaproteobacteria* | *Proteobacteria* |
| 36 | *Helicobacter* spp. | ND | *Helicobacteraceae* | [*Campylobacterales*](https://en.wikipedia.org/wiki/Campylobacterales) | [*Epsilonproteobacteria*](https://en.wikipedia.org/wiki/Epsilonproteobacteria) | *Proteobacteria* |
| 37 | *Klebsiella* spp. | ND | *Enterobacteriaceae* | *Enterobacterales* | *Gammaproteobacteria* | *Proteobacteria* |
| 38 | *Morganella* spp. | ND | [*Morganellaceae*](https://en.wikipedia.org/wiki/Morganellaceae) | *Enterobacterales* | *Gammaproteobacteria* | *Proteobacteria* |
| 39 | *Salmonella* spp. | ND | *Enterobacteriaceae* | *Enterobacterales* | *Gammaproteobacteria* | *Proteobacteria* |
| 40 | *Shigella* spp. | ND | *Enterobacteriaceae* | *Enterobacterales* | *Gammaproteobacteria* | *Proteobacteria* |
| 41 | *Yersinia* spp. | ND | [*Yersiniaceae*](https://en.wikipedia.org/wiki/Yersiniaceae) | *Enterobacterales* | *Gammaproteobacteria* | *Proteobacteria* |
| 42 | *Leptospira* spp. | ND | [*Leptospiraceae*](https://en.wikipedia.org/wiki/Leptospiraceae) | [*Leptospirales*](https://en.wikipedia.org/w/index.php?title=Leptospirales&action=edit&redlink=1) | [*Spirochaetes*](https://en.wikipedia.org/wiki/Spirochaetes) | [*Spirochaetes*](https://en.wikipedia.org/wiki/Spirochaetes) |

^1^According to WHO, 2003; Cabral, 2010; and WHO, 2017.

**Table S4.** Spearman correlation coefficients between the taxonomic richness and evenness indices. The significance of the reported coefficients was p < 0.001 (N = 230).

|  | #OTU | Observed | ACE | Chao1 | Shannon | Simpson |
| --- | --- | --- | --- | --- | --- | --- |
| #OTU | 1 | 0.95 | 0.98 | 0.92 | 0.65 | 0.45 |
| Observed |  | 1 | 0.95 | 0.98 | 0.68 | 0.51 |
| ACE |  |  | 1 | 0.94 | 0.61 | 0.41 |
| Chao1 |  |  |  | 1 | 0.65 | 0.49 |
| Shannon |  |  |  |  | 1 | 0.88 |
| Simpson |  |  |  |  |  | 1 |

Table S5. Seasonal variation of sequencing reads and alpha diversity indices in surface water; geometric mean (±standard error).

| **Parameter** | **Autumn** | **Winter** | **Spring** | **Summer** |
| --- | --- | --- | --- | --- |
| Reads | 25,500±2,330 | 23,200±2,380 | 26,100±2,230 | 22,200±1,810* |
| OTUs | 930±30 | 940±40 | 940±40 | 770±20* |
| Observed | 760±20 | 780±30 | 760±20 | 630±20* |
| ACE | 1,300±40 | 1,300±50 | 1,300±50 | 1,040±30* |
| Chao1 | 950±21 | 990±30 | 960±30 | 790±20* |
| Shannon | 4.9±0.0 | 5.0±0.0 | 4.9±0.0 | 4.8±0.0* |
| Simpson | 0.98±0.0 | 0.98±0.0 | 0.98±0.0 | 0.97±0.0* |

*Total number of samples included = 115. The parameter values were significantly lower in summer as compared to other seasons (p < 0.05). There were no significant differences in the parameter values between the autumn, winter and spring.

Table S6. Top 3 highest sample prevalence OTUs in each sample group.

| **Sample group** | **OTU** | **Sample prevalence (%)** | **Taxonomic identification** |
| --- | --- | --- | --- |
| All samples (n=230) | 560655 | 54 | *Pelagibacteraceae*;f_*Alphaproteobacteria*;c |
|  | 1106956 | 53 | *Methylophilaceae*;f_*Betaproteobacteria*;c |
|  | 848585 | 52 | *Comamonadaceae*;f_ *Betaproteobacteria*;c |
| Sewage; influent (n=7) | 776733 | 100 | *Campylobacteraceae*;f_ *Epsilonproteobacteria*;c |
|  | 1109080 | 100 | *Campylobacteraceae*;f_ *Epsilonproteobacteria*;c |
|  | 825090 | 100 | *Campylobacteraceae*;f_ *Epsilonproteobacteria*;c |
|  | 839376 | 100 | *Aeromonadaceae*;f_ *Gammaproteobacteria*;c |
| Sewage; effluent (n=52) | 776733 | 83 | *Campylobacteraceae*;f_ *Epsilonproteobacteria*;c |
|  | 1106940 | 73 | *Comamonadaceae*;f_ *Betaproteobacteria*;c |
|  | 334424 | 73 | *Moraxellaceae*;f_ *Gammaproteobacteria*;c |
| Industrial effluent (n=15) | 1092833 | 75 | *Weeksellaceae*;f_ *Bacteroidetes*;p |
|  | 255518 | 56 | *Comamonadaceae*;f_ *Betaproteobacteria*;c |
|  | 1092718 | 50 | *Weeksellaceae*;f_ *Bacteroidetes*;p |
|  | 554390 | 50 | *Bacteriovoracaceae*;f_ *Deltaproteobacteria*;c |
| Mine runoff (n=4) | 6494 | 75 | *Gallionellaceae*;f_ *Betaproteobacteria*;c |
|  | 1106940 | 75 | *Comamonadaceae*;f_ *Betaproteobacteria*;c |
|  | 829669 | 50 | *Comamonadaceae*;f_ *Betaproteobacteria*;c |
|  | 1110291 | 50 | *Oxalobacteraceae*;f_ *Betaproteobacteria*;c |
| Surface water (n=115) | 560655 | 96 | *Pelagibacteraceae*;f_*Alphaproteobacteria*;c |
|  | 1106956 | 98 | *Methylophilaceae*;f_*Betaproteobacteria*;c |
|  | 848585 | 96 | *Comamonadaceae*;f_ *Betaproteobacteria*;c |
| Pretreated water (n=10) | 625083 | 100 | *Chitinophagaceae*;f_ *Bacteroidetes*;p |
|  | 806744 | 100 | *Oxalobacteraceae*;f_ *Bacteroidetes*;p |
|  | 723936 | 100 | *Oxalobacteraceae*;f_ *Bacteroidetes*;p |
|  | 560655 | 100 | *Pelagibacteraceae*;f_*Alphaproteobacteria*;c |
|  | 1106956 | 100 | *Comamonadaceae*;f_ *Betaproteobacteria*;c |
| Groundwater observation well (n=16) | 592524 | 76 | mb2424;f_ *Acidobacteria*;p |
|  | 923760 | 76 | NC;f_ *Betaproteobacteria*;c |
|  | 832194 | 71 | *Rhodospirillaceae*;f_ *Alphaproteobacteria*;c |
| Production well (n=10) | 571360 | 100 | *Rhodospirillaceae*;f_ *Alphaproteobacteria*;c |
|  | 583130 | 100 | *Acetobacteraceae*;f_ *Alphaproteobacteria*;c |
|  | 832194 | 100 | *Rhodospirillaceae*;f_ *Alphaproteobacteria*;c |
|  | 756153 | 100 | NC;f_ Betaproteobacteria;c |
|  | 1109401 | 100 | NC;f_ *Betaproteobacteria*;c |
|  | 203879 | 100 | NC;f_ *Betaproteobacteria*;c |
|  | 220761 | 100 | NC;f_ *Betaproteobacteria*;c |

n, number of samples. The relative abundance of each core OTUs were > 0.01% of the total read counts of that particular sample. The core communities of *Proteobacteria* phylum were classified into family and class level. The other taxa were classified into family and phylum level. NC= not classified.

Table S7. Seasonal variation of mean and standard error KO hits (log_10_ N).

| Predicted Function | Yearly mean (N=230) | Autumn (n=70) | Winter (n=54) | Spring (n=61) | Summer (n=45) | **Dunn’s post-hoc test (p*)** | Significance |
| --- | --- | --- | --- | --- | --- | --- | --- |
| Amino Acid Metabolism | 5.13±0.02 | 5.13±0.02 | 5.21±0.06 | 5.00±0.03 | 5.19±0.07 | 0.020;  0.021;  0.013 | Autumn>Spring;  Winter>Spring;  Summer>Spring |
| Carbohydrate Metabolism | 5.05±0.02 | 5.09±0.02 | 5.13±0.06 | 4.94±0.03 | 5.06±0.07 | 0.004;  0.039 | Autumn>Spring;  Winter>Spring |
| Lipid and Fatty Acid Metabolism | 4.66±0.02 | 4.66±0.02 | 4.73±0.06 | 4.53±0.03 | 4.73±0.07 | 0.022;  0.029;  0.006 | Autumn>Spring;  Winter>Spring;  Summer>Spring |
| Energy Metabolism | 4.72±0.02 | 4.74±0.02 | 4.81±0.06 | 4.60±0.03 | 4.77±0.07 | 0.009;  0.009 | Autumn>Spring;  Winter>Spring |
| Nitrogen Metabolism | 3.74±0.03 | 3.73±0.25 | 3.84±0.07 | 3.60±0.04 | 3.81±0.07 | 0.019;  0.007;  0.005 | Autumn>Spring;  Summer>Spring;  Winter>Spring |
| Sulfur Metabolism | 4.06±0.02 | 4.03±0.02 | 4.17±0.06 | 3.92±0.04 | 4.16±0.07 | 0.051;  0.003;  0.002 | Autumn>Spring;  Winter>Spring;  Summer>Spring |
| Methane Metabolism | 3.90±0.03 | 3.92±0.03 | 4.05±0.07 | 3.75±0.04 | 3.90±0.07 | 0.006;  <0.001 | Autumn>Spring;  Winter>Spring |
| Amino Sugar and Nucleotide Sugar Metabolism | 3.91±0.02 | 3.92±0.02 | 4.00±0.06 | 3.78±0.03 | 3.94±0.07 | 0.01;  0.005 | Autumn>Spring;  Winter>Spring |
| Glycan Biosynthesis and Metabolism | 3.99±0.02 | 4.02±0.02 | 4.07±0.06 | 3.86±0.04 | 4.03±0.07 | 0.005;  0.017;  0.022 | Autumn>Spring;  Winter>Spring;  Summer>Spring |
| Pyrimidine Metabolism | 4.08±0.02 | 4.08±0.02 | 4.19±0.06 | 3.95±0.03 | 4.12±0.07 | 0.016;  0.003 | Autumn>Spring;  Winter>Spring |
| Metabolism of cofactors and Vitamins | 4.93±0.02 | 4.93±0.02 | 5.03±0.06 | 4.80±0.03 | 4.98±0.07 | 0.015;  0.004;  0.011 | Autumn>Spring;  Winter>Spring;  Summer>Spring |
| Nucleotide metabolism | 4.35±0.02 | 4.36±0.02 | 4.41±0.06 | 4.23±0.04 | 4.44±0.07 | 0.020;  0.006;  0.048 | Autumn>Spring;  Summer>Spring;  Winter>Spring |
| Metabolism of Terpenoides and Polyketides | 4.07±0.02 | 4.11±0.02 | 4.16±0.06 | 3.96±0.03 | 4.08±0.07 | 0.005;  0.010 | Autumn>Spring;  Winter>Spring |
| Biosynthesis of Secondary Metabolites | 3.96±0.02 | 3.99±0.02 | 4.02±0.06 | 3.85±0.03 | 3.99±0.07 | 0.030 | Autumn>Spring |
| Xenobiotics Metabolism | 4.42±0.02 | 4.43±0.25 | 4.51±0.07 | 4.30±0.03 | 4.43±0.25 | 0.009;  0.021 | Summer>Spring;  Autumn>Spring |
| Unclassified Metabolism | 5.15±0.02 | 5.17±0.02 | 5.23±0.06 | 5.03±0.03 | 5.18±0.07 | 0.008;  0.025;  0.031 | Autumn>Spring;  Winter>Spring;  Summer>Spring |
| Signaling and cellular process | 5.49±0.02 | 5.48±0.02 | 5.58±0.06 | 5.36±0.03 | 5.56±0.07 | 0.029;  0.010;  0.007 | Autumn>Spring;  Winter>Spring;  Summer>Spring |
| Environmental Information processing | 4.65±0.02 | 4.64±0.03 | 4.73±0.07 | 4.52±0.03 | 4.72±0.07 | 0.036;  0.009;  0.013 | Autumn>Spring;  Summer>Spring;  Winter>Spring |
| Genetic Information processing | 5.51±0.02 | 5.50±0.23 | 5.60±0.06 | 5.38±0.34 | 5.57±0.07 | 0.025;  0.006;  0.007 | Autumn>Spring;  Winter>Spring;  Summer>Spring |
| Health Related | 4.56±0.02 | 4.55±0.02 | 4.64±0.06 | 4.44±0.03 | 4.64±0.07 | 0.035;  0.012;  0.005 | Autumn>Spring;  Winter>Spring;  Summer>Spring |
| Unknown Function | 4.81±0.02 | 4.81±0.02 | 4.90±0.06 | 4.68±0.03 | 4.88±0.07 | 0.021;  0.009;  0.006 | Autumn>Spring;  Winter>Spring;  Summer>Spring |
| Not listed | 4.09±0.02 | 4.13±0.02 | 4.17±0.07 | 3.97±0.03 | 4.10±0.07 | 0.002;  0.026 | Autumn>Spring;  Winter>Spring |
| Total KO hits | 6.13±0.02 | 6.14±0.02 | 6.22±0.06 | 6.01±0.03 | 6.18±0.07 | 0.016;  0.014;  0.016 | Autumn>Spring;  Winter>Spring;  Summer>Spring |

***** The p-value of Dunn’s post-hoc test is without Bonferroni correction.

Table S8. Seasonal variation on PHRB read counts in surface water [median (upper-lower confidence interval)].

| **PHRB genera** | **Autumn** | **Winter** | **Spring** | **Summer** | **Dunn’s post-hoc test (p)** | **Significance** |
| --- | --- | --- | --- | --- | --- | --- |
| *Pseudomonas* | 19  (13-41) | 16  (10-39) | 42  (29-72) | 3  (2-6) | 0.01;  0.01;  0.01 | Summer< Autumn;  Summer<Spring;  Summer <Winter |
| *Mycobacterium* | 46  (37-63) | 64  (50-123) | 66  (41-89) | 26  (18-33) | 0.03;  0.01;  0.01 | Summer< Autumn;  Summer<Spring;  Summer <Winter |
| *Acinetobacter* | 6  (1-10) | 10  (4-16) | 4  (2-8) | 1  (1-4) | 0.05*;  0.01;  0.04 | Summer<Autumn;  Summer<Winter;  Summer<Spring |
| *Bacteroides* | 2  (0-4) | 10  (6-14) | 7  (4-14) | 1  (0-1) | 0.01;  0.01;  0.01;  0.01 | Summer<Spring; Summer<Winter;  Autumn<Spring;  Autumn<Winter |
| *Clostridium* | 1  (0-2) | 6  ( 2-10) | 2  (2-5) | 0 | 0.01;  0.01;  0.03;  0.01 | Summer<Spring;  Summer<Winter;  Autumn<Spring;  Autumn<Winter |
| *Legionella* | 1  (0-2) | 1  (1-2) | 1  (0-1) | 0  (0-1) | - | - |
| *Prevotella* | 0  (0-1) | 1  (0-1) | 1  (0-2) | 0 | 0.01;  0.01;  0.05* | Summer< Autumn;  Summer<Spring;  Autumn< Spring |
| *Desulfovibrio* | 0  (0-1) | 0  (0-1) | 0  (0-1) | 0 | 0.01;  0.09*;  0.05* | Summer<Spring;  Summer<Winter;  Summer<Autumn |
| *Citrobacter* | 0 | 0 | 0  (0-1) | 2  (0-9) | 0.01;  0.01;  0.04 | Winter<Summer;  Autumn<Summer;  Spring<Summer |
| *Bifidobacterium* | 0 | 1  (0-2) | 0  (0-1) | 0 | 0.01;  0.01;  0.13*;  0.01 | Summer<Spring;  Summer<Winter;  Autumn<spring;  Autumn<Winter |
| *Lactococcus* | 0 | 0  (0-1) | 0 | 0 | 0.03;  0.12*;  0.16* | Summer<Winter;  Summer<Spring;  Autumn<Winter |
| *Ruminococcus* | 0 | 0  (0-1) | 0 | 0 | 0.03;  0.12* | Autumn<Winter;  Summer<Winter |
| *Enterococcus* | 0 | 0 | 0  (0-1) | 0 | 0.02;  0.02 | Summer<Spring;  Winter<Spring |
| Total PHRB reads | 102  (85-131) | 163  (100-255) | 152  (123-246) | 44  (36-67) | 0.01;  0.01;  0.01;  0.17* | Summer<Autumn;  Summer<Winter;  Summer<Spring;  Autumn<Spring |
| The seasonal pairs marked with * had significant difference only before Bonferroni correction. | | | | | | |


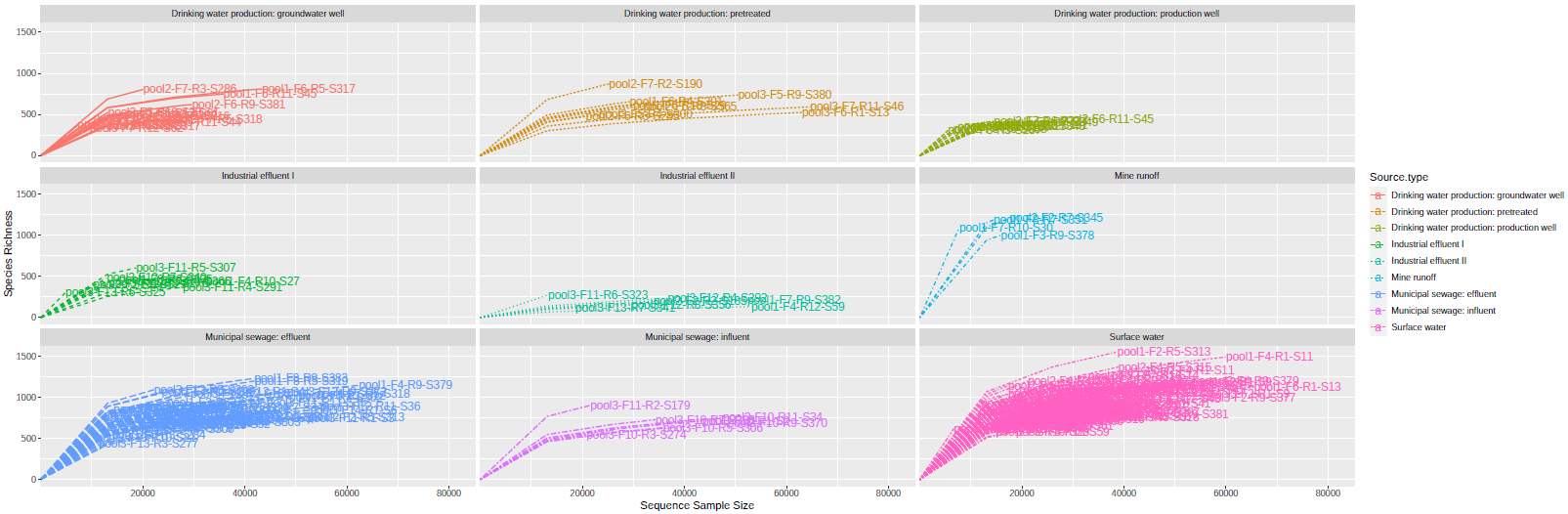


Figure S1. Rarefaction curves of the bacterial microbial communities based on 16S rRNA gene sequences from different water sample groups.





Figure S2. Box-plot comparison of (A) read counts and alpha-diversity indices: (B) Chao1, (C) Shannon diversity index and (D) Simpson diversity index of bacterial communities in the sample groups.


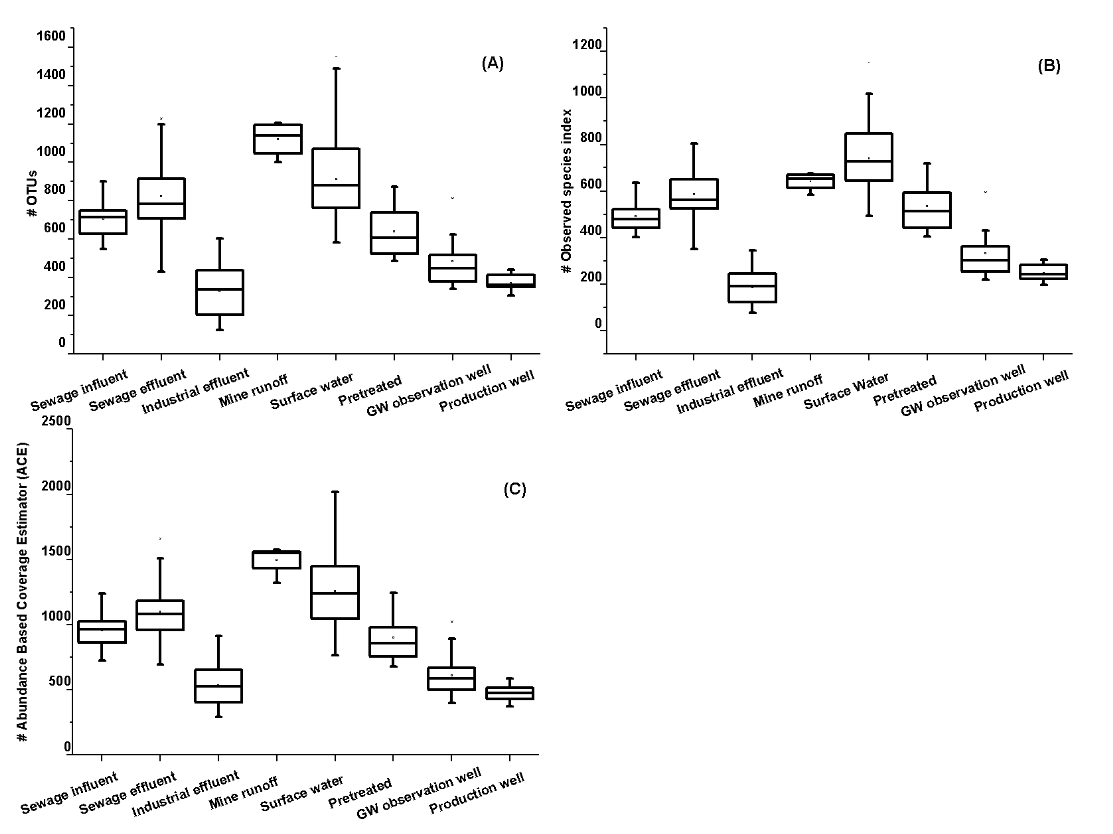


Figure S3. Box-plot comparison of (A) the number of OTUs, (B) observed species index and (C) ACE estimator of bacterial communities in the sample groups.

# .


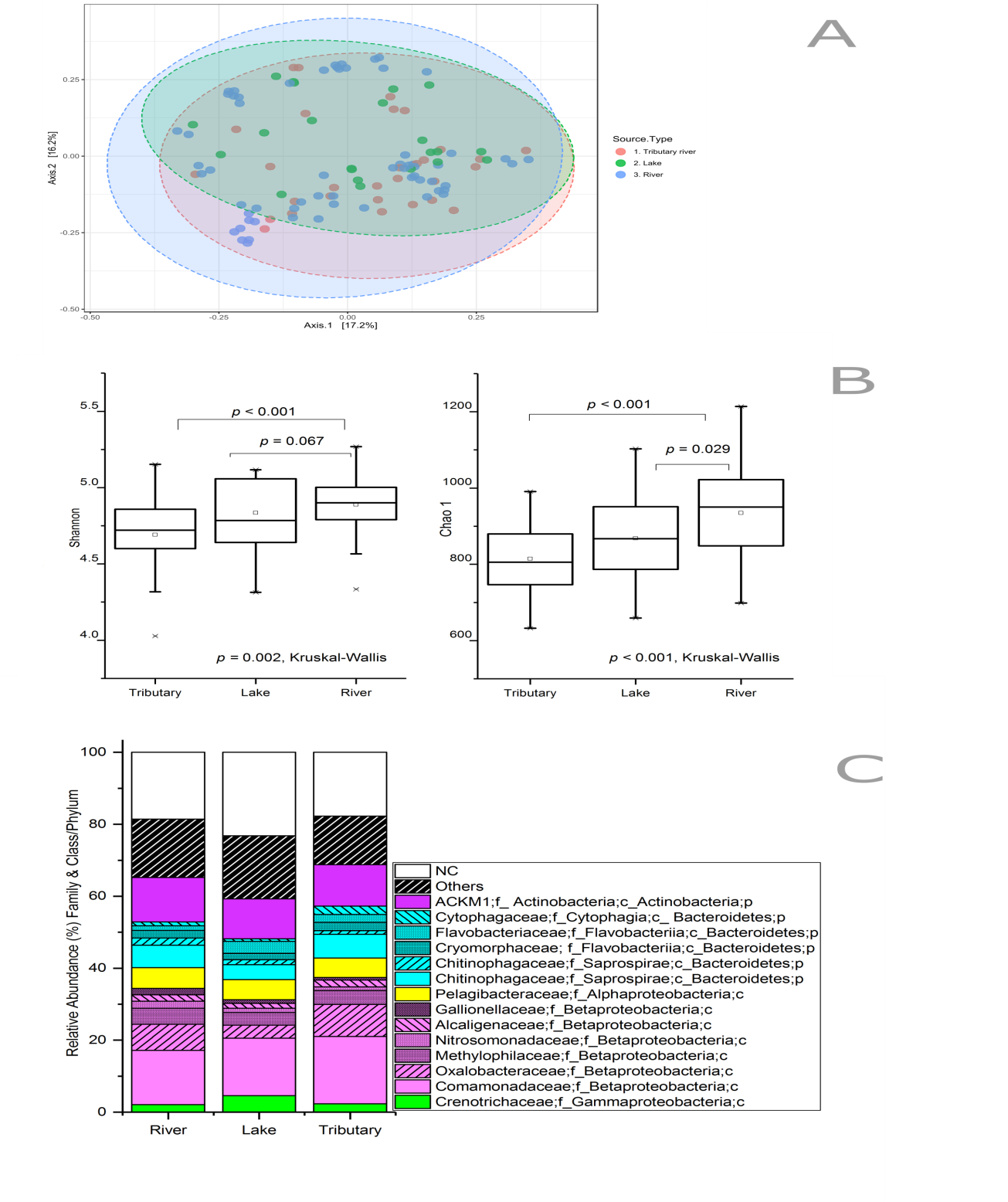


# Figure S4. Bacterial communities in the surface water sample sub-groups of Tributary River, Lake Region and River region. A. Nonmetric multidimensional scaling and analysis of dissimilarities between bacterial communities in the subgroups [ANOSIM] R = 0.10; *p*-value < 0.002 [NMDS]. B. Box-plot comparison of Shannon and Chao1 values. C. Bacterial taxonomic structure at family level in the surface water sub-groups. Others: Families having read contribution more than 2% in at least in one sample type are shown in figure otherwise grouped as others. NC: Not classified.


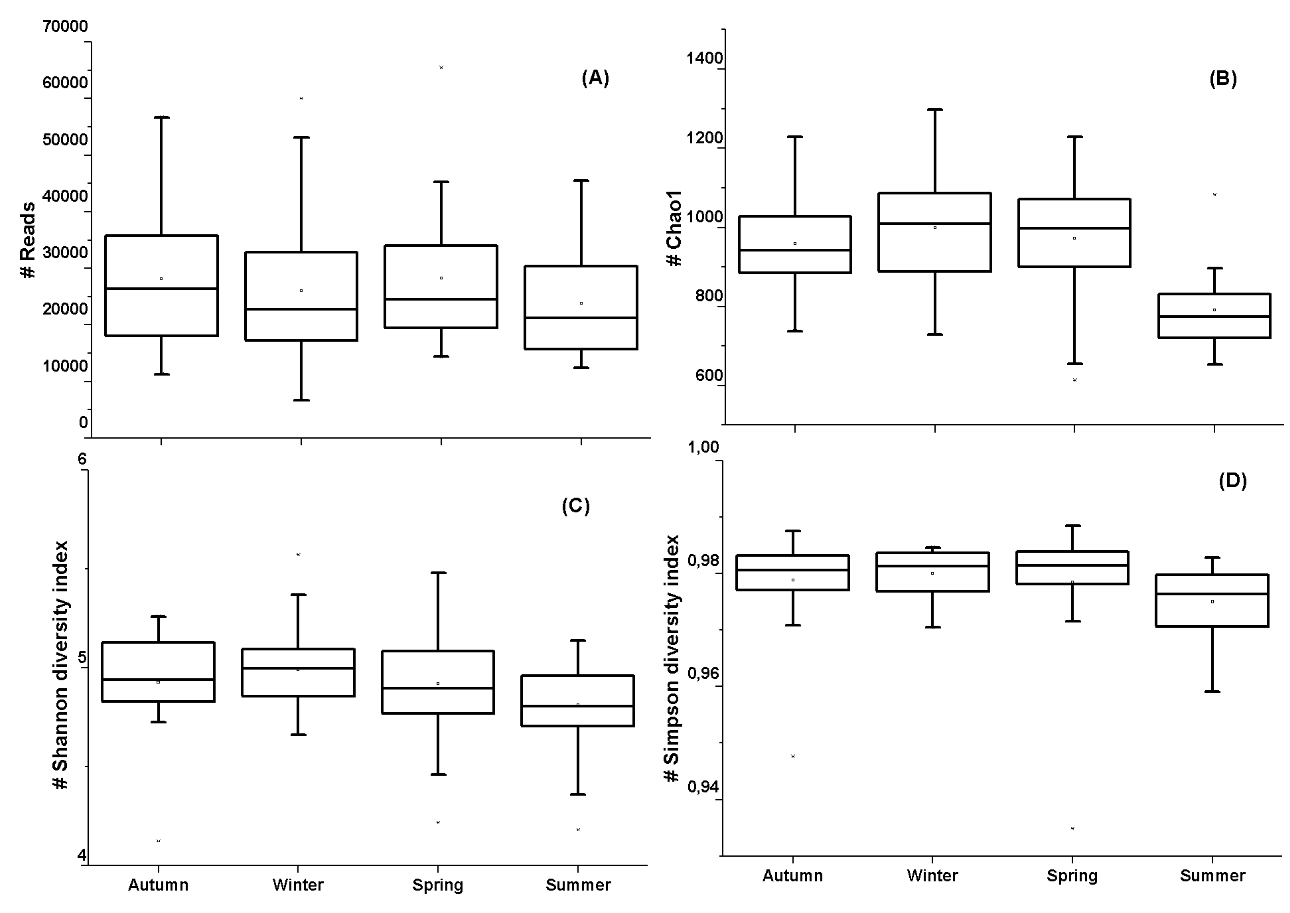


Figure S5. Box-plot comparison of (A) read counts and alpha-diversity indices: (B) Chao1, (C) Shannon and (D) Simpson of bacterial communities in surface water in four seasons of a year.

**

**

Figure S6. Box-plot comparison of (A) the number of OTUs, (B) observed species index and (C) ACE estimator of bacterial communities in surface water in four seasons of a year.

#
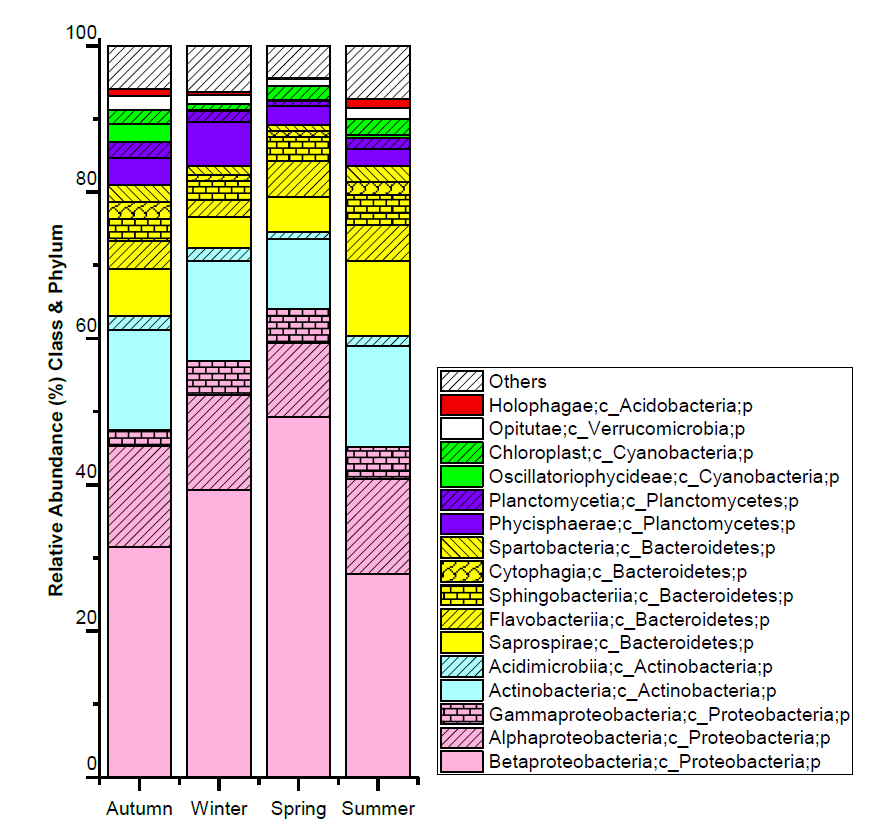
Figure S7. Seasonal variation in the bacterial taxa in surface water samples. Others: Taxa with relative abundance less than 1%.

**References**

1. Cabral J.P.S, 2010. Water Microbiology. Bacterial Pathogens and Water. *Int. J. Environ. Res. Public Health,* 7, 10, 3657-3703. doi: 10.3390/ijerph7103657.
2. WHO, 2017. World Health Organization. Guidelines for drinking-water quality: fourth edition incorporating the first addendum. ISBN 978-92-4-154995-0, Geneva, Switzerland.
3. WHO, 2003. World Health Organization. Guidelines for safe recreational water environments. Volume 1, Coastal and fresh waters. Chapter 4: Faecal pollution and water quality, 51-101, Geneva, Switzerland.
