# Supplementary material for: Bacterial diversity and predicted enzymatic function in a multipurpose surface water system – from wastewater effluent discharges to drinking water production
Source: Environ Microbiome. 2021 May 22;16:11. doi: 10.1186/s40793-021-00379-w (PMC8140503; doi:10.1186/s40793-021-00379-w)
Supplement: Supplementary file 2 — Additional file 2: Supplemental Data Sheet: Core bacterial communities. Supplemental Data Sheet S1. Municipal influent. Supplemental Data Sheet S2. Municipal effluent. Supplemental Data Sheet S3. Industrial effluent I. Supplemental Data Sheet S4. Industrial effluent II. Supplemental Data Sheet S5. Mine runoff. Supplemental Data Sheet S6. Surface water. Supplemental Data Sheet S7. Pretreated water. Supplemental Data Sheet S8. Groundwater (combine groundwater observation and production wells). Supplemental Data Sheet S9. Groundwater observation wells. Supplemental Data Sheet S10. Groundwater production wells. [file 40793_2021_379_MOESM2_ESM.pdf]

Supplemental Data Sheets S1-S10. Core bacterial communities in the groups of water samples

Bacterial diversity and predicted enzymatic function in a multipurpose surface water system – from wastewater effluent discharges to drinking water production

by Ananda Tiwari, Anna-Maria Hokajärvi, Jorge Santo Domingo, Michael Elk, Balamuralikrishna Jayaprakash, Hodon Ryu, Sallamaari Siponen, Asko Vepsäläinen,
Ari Kauppinen, Osmo Puurunen, Aki Artimo, Noora Perkola, Timo Huttula, Ilkka T. Miettinen and Tarja Pitkänen

Selection criteria for Supplemental Data Sheets:
1. Detection frequency of an OTU ≥ 20% in the sample group (sample prevalence; the OTU was found in 20% of samples in the specified sample group)
2. Relative abundance of an OTU ≥ 0.01 % of total reads within each sample.

The OTUs having top 2-3 highest sample prevalence in each group are highlighted with red color.

Supplemental Data Sheet S1. Core bacterial communities in the municipal influent (n=7).

OTU	Detection Frequency %	Phylum	Class	Order	Family	Genus
1109857	86	Proteobacteria	Betaproteobacteria	Burkholderiales	Comamonadaceae	Comamonas
942142	29	Proteobacteria	Betaproteobacteria	Burkholderiales	Comamonadaceae	Polaromonas
776733	100	Proteobacteria	Epsilonproteobacteria	Campylobacterales	Campylobacteraceae	Arcobacter
1109080	100	Proteobacteria	Epsilonproteobacteria	Campylobacterales	Campylobacteraceae	Arcobacter
825090	100	Proteobacteria	Epsilonproteobacteria	Campylobacterales	Campylobacteraceae	Arcobacter
807542	71	Proteobacteria	Epsilonproteobacteria	Campylobacterales	Campylobacteraceae	Arcobacter
4400334	43	Proteobacteria	Epsilonproteobacteria	Fusobacteriales	Leptotrichiaceae	Leptotrichia
11164	29	Proteobacteria	Epsilonproteobacteria	Campylobacterales	Campylobacteraceae	Arcobacter
697170	29	Proteobacteria	Epsilonproteobacteria	Lactobacillales	Carnobacteriaceae	Trichococcus
546186	29	Proteobacteria	Epsilonproteobacteria	Campylobacterales	Campylobacteraceae	Arcobacter
839376	100	Proteobacteria	Gammaproteobacteria	Aeromonadales	Aeromonadaceae	
1074011	86	Proteobacteria	Gammaproteobacteria	Pseudomonadales	Moraxellaceae	Acinetobacter
542450	86	Proteobacteria	Gammaproteobacteria	Pseudomonadales	Moraxellaceae	Acinetobacter
1061335	43	Proteobacteria	Gammaproteobacteria	Pseudomonadales	Moraxellaceae	Acinetobacter
1110300	43	Proteobacteria	Gammaproteobacteria	Aeromonadales	Aeromonadaceae	
1121651	29	Proteobacteria	Gammaproteobacteria	Aeromonadales	Aeromonadaceae	
607810	29	Proteobacteria	Gammaproteobacteria	Fusobacteriales		

**Supplemental Data Sheet S2.** Core bacterial communities in the municipal effluent (n=52).						
**OTU**	**Detection Frequency %**	**Phylum**	**Class**	**Order**	**Family**	**Genus**
781203	25	Bacteroidetes	Saprospirae	[Saprospirales]	Chitinophagaceae	Sediminibacterium
4322041	21	Bacteroidetes	Saprospirae	[Saprospirales]	Chitinophagaceae	Sediminibacterium
1106940	73	Proteobacteria	Betaproteobacteria	Burkholderiales	Comamonadaceae	
609943	65	Proteobacteria	Betaproteobacteria	Procabacteriales	Procabacteriaceae	
796924	60	Proteobacteria	Betaproteobacteria	Burkholderiales	Comamonadaceae	Rhodoferax
94716	38	Proteobacteria	Betaproteobacteria	Procabacteriales	Procabacteriaceae	
155812	37	Proteobacteria	Betaproteobacteria	Procabacteriales	Procabacteriaceae	
829669	33	Proteobacteria	Betaproteobacteria	Burkholderiales	Comamonadaceae	Hydrogenophaga
1109857	31	Proteobacteria	Betaproteobacteria	Burkholderiales	Comamonadaceae	Comamonas
942142	31	Proteobacteria	Betaproteobacteria	Burkholderiales	Comamonadaceae	Polaromonas
818376	29	Proteobacteria	Betaproteobacteria	Burkholderiales	Comamonadaceae	Rhodoferax
1123672	27	Proteobacteria	Betaproteobacteria	Burkholderiales	Oxalobacteraceae	
835100	23	Proteobacteria	Betaproteobacteria	Burkholderiales	Comamonadaceae	Polaromonas
776733	83	Proteobacteria	Epsilonproteobacteria	Campylobacterales	Campylobacteraceae	Arcobacter
1109080	54	Proteobacteria	Epsilonproteobacteria	Campylobacterales	Campylobacteraceae	Arcobacter
807542	48	Proteobacteria	Epsilonproteobacteria	Campylobacterales	Campylobacteraceae	Arcobacter
825090	40	Proteobacteria	Epsilonproteobacteria	Campylobacterales	Campylobacteraceae	Arcobacter
334424	73	Proteobacteria	Gammaproteobacteria	Pseudomonadales	Moraxellaceae	
586540	38	Proteobacteria	Gammaproteobacteria	Pseudomonadales	Moraxellaceae	
348203	25	Proteobacteria	Gammaproteobacteria	Pseudomonadales	Moraxellaceae	
839376	25	Proteobacteria	Gammaproteobacteria	Aeromonadales	Aeromonadaceae	
238484	21	Proteobacteria	Gammaproteobacteria	Pseudomonadales	Moraxellaceae	

**Supplemental Data Sheet S3.** Core bacterial communities in the industrial effluent I (n=6).						
**OTU**	**Detection Frequency %**	**Phylum**	**Class**	**Order**	**Family**	**Genus**
572010	33	Bacteroidetes	Bacteroidia	Bacteroidales	Bacteroidaceae	Bacteroides
45562	22	Bacteroidetes	Bacteroidia	Bacteroidales	Porphyromonadaceae	
459820	22	Bacteroidetes	Bacteroidia	Bacteroidales	Porphyromonadaceae	Paludibacter
1092833	56	Bacteroidetes	Flavobacteriia	Flavobacteriales	[Weeksellaceae]	Cloacibacterium
1092718	33	Bacteroidetes	Flavobacteriia	Flavobacteriales	[Weeksellaceae]	Cloacibacterium
1041552	22	Bacteroidetes	Flavobacteriia	Flavobacteriales	[Weeksellaceae]	Cloacibacterium
831485	22	Bacteroidetes	Flavobacteriia	Flavobacteriales	Flavobacteriaceae	Flavobacterium
818174	22	Bacteroidetes	Saprospirae	[Saprospirales]	Saprospiraceae	
825904	22	Bacteroidetes	Sphingobacteriia	Sphingobacteriales		
41211	33	Chloroflexi	Chloroflexi	[Roseiflexales]	[Kouleothrixaceae]	Kouleothrix
255811	33	Chloroflexi	Chloroflexi	[Roseiflexales]	[Kouleothrixaceae]	Kouleothrix
249034	22	Proteobacteria	Alphaproteobacteria			
4408777	22	Proteobacteria	Alphaproteobacteria	Caulobacterales	Caulobacteraceae	Asticcacaulis
341537	22	Proteobacteria	Alphaproteobacteria	Sphingomonadales	Sphingomonadaceae	Novosphingobium
1007128	56	Proteobacteria	Betaproteobacteria	Rhodocyclales	Rhodocyclaceae	Zoogloea
835093	44	Proteobacteria	Betaproteobacteria	Rhodocyclales	Rhodocyclaceae	Zoogloea
864150	33	Proteobacteria	Betaproteobacteria	Rhodocyclales	Rhodocyclaceae	Zoogloea
279025	33	Proteobacteria	Betaproteobacteria	Burkholderiales	Comamonadaceae	
1109857	33	Proteobacteria	Betaproteobacteria	Burkholderiales	Comamonadaceae	Comamonas
707172	33	Proteobacteria	Betaproteobacteria	Burkholderiales	Comamonadaceae	Limnohabitans
2105446	33	Proteobacteria	Betaproteobacteria	Procabacteriales	Procabacteriaceae	
4298844	33	Proteobacteria	Betaproteobacteria	Procabacteriales	Procabacteriaceae	
1106324	22	Proteobacteria	Betaproteobacteria	Burkholderiales	Comamonadaceae	
255518	22	Proteobacteria	Betaproteobacteria	Burkholderiales	Comamonadaceae	
559109	22	Proteobacteria	Betaproteobacteria	Procabacteriales	Procabacteriaceae	
94716	22	Proteobacteria	Betaproteobacteria	Procabacteriales	Procabacteriaceae	
1111128	22	Proteobacteria	Betaproteobacteria	Burkholderiales	Comamonadaceae	Hydrogenophaga
647775	22	Proteobacteria	Betaproteobacteria	Burkholderiales	Comamonadaceae	
829023	22	Proteobacteria	Betaproteobacteria	Burkholderiales	Comamonadaceae	
702854	22	Proteobacteria	Betaproteobacteria	Burkholderiales	Comamonadaceae	Pelomonas
554390	22	Proteobacteria	Deltaproteobacteria	Bdellovibrionales	Bacteriovoracaceae	Peredibacter
1109887	22	Proteobacteria	Epsilonproteobacteria	Campylobacterales	Campylobacteraceae	Sulfurospirillum
367605	78	Proteobacteria	Gammaproteobacteria	HOC36		
100064	67	Proteobacteria	Gammaproteobacteria	Thiotrichales	Thiotrichaceae	Thiothrix
1110300	56	Proteobacteria	Gammaproteobacteria	Aeromonadales	Aeromonadaceae	
831272	44	Proteobacteria	Gammaproteobacteria	Thiotrichales	Thiotrichaceae	Thiothrix
853549	33	Proteobacteria	Gammaproteobacteria	Aeromonadales	Aeromonadaceae	
586481	22	Proteobacteria	Gammaproteobacteria	Thiotrichales	Thiotrichaceae	Thiothrix
1098706	22	Proteobacteria	Gammaproteobacteria	Alteromonadales	[Chromatiaceae]	Rheinheimera
739890	22	Proteobacteria	Gammaproteobacteria	Pseudomonadales	Moraxellaceae	Acinetobacter
1134828	22	SR1				

**Supplemental Data Sheet S4.** Core bacterial communities in the industrial effluent II (n=9).						
**OTU**	**Detection Frequency %**	**Phylum**	**Class**	**Order**	**Family**	**Genus**
1092833	100	Bacteroidetes	Flavobacteriia	Flavobacteriales	[Weeksellaceae]	Cloacibacterium
1092718	71	Bacteroidetes	Flavobacteriia	Flavobacteriales	[Weeksellaceae]	Cloacibacterium
486302	71	Bacteroidetes	Flavobacteriia	Flavobacteriales	[Weeksellaceae]	Cloacibacterium
4154872	57	Bacteroidetes	Flavobacteriia	Flavobacteriales	[Weeksellaceae]	Cloacibacterium
1041552	57	Bacteroidetes	Flavobacteriia	Flavobacteriales	[Weeksellaceae]	Cloacibacterium
4449324	43	Bacteroidetes	Flavobacteriia	Flavobacteriales	[Weeksellaceae]	Cloacibacterium
388958	43	Bacteroidetes	Flavobacteriia	Flavobacteriales	[Weeksellaceae]	Cloacibacterium
726608	57	Proteobacteria	Alphaproteobacteria	Rhodobacterales	Rhodobacteraceae	Rhodobacter
521994	29	Proteobacteria	Alphaproteobacteria	Rhodobacterales	Rhodobacteraceae	Rhodobacter
255518	100	Proteobacteria	Betaproteobacteria	Burkholderiales	Comamonadaceae	
668923	86	Proteobacteria	Betaproteobacteria	Burkholderiales	Comamonadaceae	Schlegelella
280799	71	Proteobacteria	Betaproteobacteria	Burkholderiales	Comamonadaceae	Tepidimonas
554390	86	Proteobacteria	Deltaproteobacteria	Bdellovibrionales	Bacteriovoracaceae	Peredibacter

**Supplemental Data Sheet S5.** Core bacterial communities in the mine runoff (n=4).						
**OTU**	**Detection Frequency %**	**Phylum**	**Class**	**Order**	**Family**	**Genus**
1106943	25	Actinobacteria	Actinobacteria	Actinomycetales	Microbacteriaceae	Salinibacterium
799109	25	Bacteroidetes	Cytophagia	Cytophagales	Cytophagaceae	Leadbetterella
612723	25	Bacteroidetes	Cytophagia	Cytophagales	Cytophagaceae	Flectobacillus
1018521	25	Bacteroidetes	Sphingobacteriia	Sphingobacteriales	Sphingobacteriaceae	Pedobacter
1085831	25	Bacteroidetes	Sphingobacteriia	Sphingobacteriales	Sphingobacteriaceae	Pedobacter
1012361	25	Bacteroidetes	Sphingobacteriia	Sphingobacteriales	Sphingobacteriaceae	Pedobacter
1110257	25	Bacteroidetes	Sphingobacteriia	Sphingobacteriales	Sphingobacteriaceae	Pedobacter
34401	25	OP3	koll11	GIF10		
724604	25	OP3	koll11	GIF10		
512654	25	OP3	PBS-25			
960877	25	Proteobacteria	Alphaproteobacteria	Sphingomonadales	Sphingomonadaceae	Novosphingobium
402657	25	Proteobacteria	Alphaproteobacteria	Rhodospirillales	Rhodospirillaceae	
6494	75	Proteobacteria	Betaproteobacteria	Gallionellales	Gallionellaceae	Gallionella
1106940	75	Proteobacteria	Betaproteobacteria	Burkholderiales	Comamonadaceae	
829669	50	Proteobacteria	Betaproteobacteria	Burkholderiales	Comamonadaceae	Hydrogenophaga
1110291	50	Proteobacteria	Betaproteobacteria	Burkholderiales	Oxalobacteraceae	
1107523	50	Proteobacteria	Betaproteobacteria	Gallionellales	Gallionellaceae	Gallionella
1111096	25	Proteobacteria	Betaproteobacteria	Burkholderiales	Comamonadaceae	Rhodoferax
789011	25	Proteobacteria	Betaproteobacteria	Burkholderiales	Oxalobacteraceae	Polynucleobacter
593436	25	Proteobacteria	Betaproteobacteria	Gallionellales	Gallionellaceae	Gallionella
594332	25	Proteobacteria	Betaproteobacteria	Gallionellales	Gallionellaceae	Gallionella
1105719	25	Proteobacteria	Betaproteobacteria	Burkholderiales	Comamonadaceae	Rhodoferax
991503	25	Proteobacteria	Betaproteobacteria	Burkholderiales	Oxalobacteraceae	
832674	25	Proteobacteria	Betaproteobacteria	Burkholderiales	Oxalobacteraceae	Janthinobacterium
541363	25	Proteobacteria	Betaproteobacteria	Burkholderiales	Comamonadaceae	
574746	25	Proteobacteria	Betaproteobacteria	Gallionellales	Gallionellaceae	Gallionella
1107408	25	Proteobacteria	Betaproteobacteria	Burkholderiales	Comamonadaceae	Limnohabitans
546999	25	Proteobacteria	Betaproteobacteria	Gallionellales	Gallionellaceae	Gallionella
358252	25	Proteobacteria	Betaproteobacteria	Burkholderiales	Oxalobacteraceae	Polynucleobacter
986745	25	Proteobacteria	Betaproteobacteria	Burkholderiales	Comamonadaceae	Rhodoferax
723936	25	Proteobacteria	Betaproteobacteria	Burkholderiales	Oxalobacteraceae	Polynucleobacter
977051	25	Proteobacteria	Betaproteobacteria	Methylophilales	Methylophilaceae	
590236	25	Proteobacteria	Betaproteobacteria	Burkholderiales	Oxalobacteraceae	Janthinobacterium
818376	25	Proteobacteria	Betaproteobacteria	Burkholderiales	Comamonadaceae	Rhodoferax
572788	25	Proteobacteria	Betaproteobacteria	Burkholderiales	Oxalobacteraceae	Janthinobacterium
796924	25	Proteobacteria	Betaproteobacteria	Burkholderiales	Comamonadaceae	Rhodoferax
268413	25	Proteobacteria	Deltaproteobacteria	MBNT15		
1021984	25	Proteobacteria	Deltaproteobacteria	Myxococcales		
791566	50	Proteobacteria	Epsilonproteobacteria	Campylobacterales	Helicobacteraceae	Sulfuricurvum
811080	25	Proteobacteria	Epsilonproteobacteria	Campylobacterales	Helicobacteraceae	Sulfuricurvum
825090	25	Proteobacteria	Epsilonproteobacteria	Campylobacterales	Campylobacteraceae	Arcobacter
1094834	25	Proteobacteria	Gammaproteobacteria	Pseudomonadales	Pseudomonadaceae	Pseudomonas
3095738	25	Proteobacteria	Gammaproteobacteria	Alteromonadales	Alteromonadaceae	Cellvibrio
1110447	25	Proteobacteria	Gammaproteobacteria	Pseudomonadales	Pseudomonadaceae	Pseudomonas
670210	25	Proteobacteria	Gammaproteobacteria	Pseudomonadales	Pseudomonadaceae	Pseudomonas

Supplemental Data Sheet S6. Core bacterial communities in the surface water (n=115).

OTU	Detection Frequency %	Phylum	Class	Order	Family	Genus
737649	83	Actinobacteria	Actinobacteria	Actinomycetales	ACK-M1	
813339	69	Actinobacteria	Actinobacteria	Actinomycetales	ACK-M1	
817301	28	Actinobacteria	Actinobacteria	Actinomycetales	ACK-M1	
825494	27	Actinobacteria	Actinobacteria	Actinomycetales	ACK-M1	
806314	23	Actinobacteria	Actinobacteria	Actinomycetales	ACK-M1	
1105807	24	Bacteroidetes	Cytophagia	Cytophagales	Cytophagaceae	
664907	59	Bacteroidetes	Saprospirae	[Saprospirales]	Chitinophagaceae	Sediminibacterium
625083	59	Bacteroidetes	Saprospirae	[Saprospirales]	Chitinophagaceae	Sediminibacterium
342101	25	Bacteroidetes	Saprospirae	[Saprospirales]	Chitinophagaceae	Sediminibacterium
592846	21	Bacteroidetes	Sphingobacteriia	Sphingobacteriales		
345003	43	Chloroflexi	SL56			
28705	46	Planctomycetes	Phycisphaerae	Phycisphaerales		
36013	23	Planctomycetes	Phycisphaerae	Phycisphaerales		
4151695	48	Planctomycetes	Planctomycetia	Gemmatales	Gemmataceae	
560655	96	Proteobacteria	Alphaproteobacteria	Rickettsiales	Pelagibacteraceae	
657305	43	Proteobacteria	Alphaproteobacteria	Rhizobiales	Methylocystaceae	
228936	31	Proteobacteria	Alphaproteobacteria	Rhizobiales		
1106956	98	Proteobacteria	Betaproteobacteria	Methylophilales	Methylophilaceae	
848585	96	Proteobacteria	Betaproteobacteria	Burkholderiales	Comamonadaceae	
723936	75	Proteobacteria	Betaproteobacteria	Burkholderiales	Oxalobacteraceae	Polynucleobacter
923760	54	Proteobacteria	Betaproteobacteria			
343207	53	Proteobacteria	Betaproteobacteria	Burkholderiales	Comamonadaceae	Limnohabitans
1107408	49	Proteobacteria	Betaproteobacteria	Burkholderiales	Comamonadaceae	Limnohabitans
806744	48	Proteobacteria	Betaproteobacteria	Burkholderiales	Oxalobacteraceae	Polynucleobacter
1106940	46	Proteobacteria	Betaproteobacteria	Burkholderiales	Comamonadaceae	
593436	40	Proteobacteria	Betaproteobacteria	Gallionellales	Gallionellaceae	Gallionella
713354	36	Proteobacteria	Betaproteobacteria	Burkholderiales	Comamonadaceae	Rhodoferax
340432	33	Proteobacteria	Betaproteobacteria			
1109857	31	Proteobacteria	Betaproteobacteria	Burkholderiales	Comamonadaceae	Comamonas
730679	28	Proteobacteria	Betaproteobacteria	Burkholderiales	Alcaligenaceae	
358252	23	Proteobacteria	Betaproteobacteria	Burkholderiales	Oxalobacteraceae	Polynucleobacter
658233	45	Proteobacteria	Gammaproteobacteria	Methylococcales	Crenotrichaceae	Crenothrix
99085	37	Verrucomicrobia	Spartobacteria	[Chthoniobacterales]	[Chthoniobacteraceae]	Candidatus Xiphinematobacter

**Supplemental Data Sheet S7.** Core bacterial communities in the pretreated water (n=10).						
**OTU**	**Detection Frequency %**	**Phylum**	**Class**	**Order**	**Family**	**Genus**
813339	78	Actinobacteria	Actinobacteria	Actinomycetales	ACK-M1	
817301	78	Actinobacteria	Actinobacteria	Actinomycetales	ACK-M1	
737649	67	Actinobacteria	Actinobacteria	Actinomycetales	ACK-M1	
805542	56	Actinobacteria	Actinobacteria	Actinomycetales	ACK-M1	
578307	33	Actinobacteria	Actinobacteria	Actinomycetales		
825494	33	Actinobacteria	Actinobacteria	Actinomycetales	ACK-M1	
792520	33	Actinobacteria	Actinobacteria	Actinomycetales	ACK-M1	
806314	22	Actinobacteria	Actinobacteria	Actinomycetales	ACK-M1	
830978	22	Actinobacteria	Actinobacteria	Actinomycetales	ACK-M1	
1105807	89	Bacteroidetes	Cytophagia	Cytophagales	Cytophagaceae	
664907	67	Bacteroidetes	Saprospirae	[Saprospirales]	Chitinophagaceae	Sediminibacterium
1023749	22	Bacteroidetes	Saprospirae	[Saprospirales]	Chitinophagaceae	
625083	100	Bacteroidetes	Saprospirae	[Saprospirales]	Chitinophagaceae	Sediminibacterium
806744	100	Bacteroidetes	Saprospirae	Burkholderiales	Oxalobacteraceae	Polynucleobacter
723936	100	Bacteroidetes	Saprospirae	Burkholderiales	Oxalobacteraceae	Polynucleobacter
342101	44	Bacteroidetes	Saprospirae	[Saprospirales]	Chitinophagaceae	Sediminibacterium
592846	22	Bacteroidetes	Sphingobacteriia	Sphingobacteriales		
560655	100	Proteobacteria	Alphaproteobacteria	Rickettsiales	Pelagibacteraceae	
346466	44	Proteobacteria	Alphaproteobacteria	Burkholderiales	Comamonadaceae	
341222	22	Proteobacteria	Alphaproteobacteria	Rickettsiales	Pelagibacteraceae	
1106956	100	Proteobacteria	Betaproteobacteria	Methylophilales	Methylophilaceae	
848585	89	Proteobacteria	Betaproteobacteria	Burkholderiales	Comamonadaceae	
1109857	78	Proteobacteria	Betaproteobacteria	Burkholderiales	Comamonadaceae	Comamonas
338157	67	Proteobacteria	Betaproteobacteria	Holophagales	Holophagaceae	
593436	56	Proteobacteria	Betaproteobacteria	Gallionellales	Gallionellaceae	Gallionella
340432	44	Proteobacteria	Betaproteobacteria			
923760	33	Proteobacteria	Betaproteobacteria			
789011	33	Proteobacteria	Betaproteobacteria	Burkholderiales	Oxalobacteraceae	Polynucleobacter
1107408	33	Proteobacteria	Betaproteobacteria	Burkholderiales	Comamonadaceae	Limnohabitans
1107402	33	Proteobacteria	Betaproteobacteria	Burkholderiales	Comamonadaceae	Limnohabitans
358252	33	Proteobacteria	Betaproteobacteria	Burkholderiales	Oxalobacteraceae	Polynucleobacter
343207	33	Proteobacteria	Betaproteobacteria	Burkholderiales	Comamonadaceae	Limnohabitans
1111096	22	Proteobacteria	Betaproteobacteria	Burkholderiales	Comamonadaceae	Rhodoferax
897527	22	Proteobacteria	Betaproteobacteria	Nitrosomonadales	Nitrosomonadaceae	
557255	22	Proteobacteria	Betaproteobacteria			
730679	22	Proteobacteria	Betaproteobacteria	Burkholderiales	Alcaligenaceae	
1105353	22	Proteobacteria	Betaproteobacteria	Burkholderiales	Comamonadaceae	
713354	22	Proteobacteria	Betaproteobacteria	Burkholderiales	Comamonadaceae	Rhodoferax
546705	22	Proteobacteria	Betaproteobacteria	Burkholderiales	Oxalobacteraceae	Polynucleobacter
340581	22	Proteobacteria	Betaproteobacteria	Burkholderiales	Oxalobacteraceae	Polynucleobacter
1106940	22	Proteobacteria	Betaproteobacteria	Burkholderiales	Comamonadaceae	
658233	22	Proteobacteria	Gammaproteobacteria	Methylococcales	Crenotrichaceae	Crenothrix
209628	33	Verrucomicrobia	Opitutae	Opitutales	Opitutaceae	

Supplemental Data Sheet S8. Core bacterial communities in the groundwater observation well and production well (n=26).

OTU	Detection Frequency %	Phylum	Class	Order	Family	Genus
629448	27	Acidobacteria	Acidobacteria-5			
557143	58	Acidobacteria	Acidobacteria-6	iii1-15		
592524	54	Acidobacteria	Acidobacteria-6	iii1-15	mb2424	
582670	27	Acidobacteria	Acidobacteria-6	iii1-15		
179461	27	Bacteroidetes	Flavobacteriia	Flavobacteriales	Cryomorphaceae	Fluviicola
525642	27	Bacteroidetes	Sphingobacteriia	Sphingobacteriales		
356355	23	Planctomycetes	Planctomycetia	Planctomycetales	Planctomycetaceae	Planctomyces
832194	88	Proteobacteria	Alphaproteobacteria	Rhodospirillales	Rhodospirillaceae	
571360	81	Proteobacteria	Alphaproteobacteria	Rhodospirillales	Rhodospirillaceae	
756153	62	Proteobacteria	Alphaproteobacteria	Rhizobiales	Hyphomicrobiaceae	Rhodoplanes
583130	62	Proteobacteria	Alphaproteobacteria	Rhodospirillales	Acetobacteraceae	
569368	38	Proteobacteria	Alphaproteobacteria	Rhizobiales	Hyphomicrobiaceae	Rhodoplanes
583185	38	Proteobacteria	Alphaproteobacteria	Rhizobiales	Hyphomicrobiaceae	
716576	31	Proteobacteria	Alphaproteobacteria	Rhodospirillales	Rhodospirillaceae	
244655	31	Proteobacteria	Alphaproteobacteria	Rhodospirillales	Rhodospirillaceae	
574255	23	Proteobacteria	Alphaproteobacteria	Rhizobiales	Hyphomicrobiaceae	Hyphomicrobium
923760	81	Proteobacteria	Betaproteobacteria			
203879	77	Proteobacteria	Betaproteobacteria			
593212	73	Proteobacteria	Betaproteobacteria			
220761	62	Proteobacteria	Betaproteobacteria			
1109401	58	Proteobacteria	Betaproteobacteria			
694781	50	Proteobacteria	Betaproteobacteria	MND1		
240278	46	Proteobacteria	Betaproteobacteria			
567333	38	Proteobacteria	Betaproteobacteria			
548352	31	Proteobacteria	Betaproteobacteria	IS-44		
903951	27	Proteobacteria	Betaproteobacteria			
254098	27	Proteobacteria	Betaproteobacteria			
817446	27	Proteobacteria	Betaproteobacteria			
3984836	27	Proteobacteria	Betaproteobacteria			
536176	23	Proteobacteria	Betaproteobacteria	IS-44		
771634	31	Proteobacteria	Deltaproteobacteria	Myxococcales	0319-6G20	

Supplemental Data Sheet S9. Core bacterial communities in the groundwater observation wells (n=16).

OTU	Detection Frequency %	Phylum	Class	Order	Family	Genus
629448	29	Acidobacteria	Acidobacteria-5			
592524	76	Acidobacteria	Acidobacteria-6	iii1-15	mb2424	
557143	47	Acidobacteria	Acidobacteria-6	iii1-15		
584801	24	Acidobacteria	Acidobacteria-6	iii1-15		
4395040	24	Acidobacteria	Solibacteres	Solibacterales	[Bryobacteraceae]	
813339	24	Actinobacteria	Actinobacteria	Actinomycetales	ACK-M1	
179461	24	Bacteroidetes	Flavobacteriia	Flavobacteriales	Cryomorphaceae	Fluviicola
625083	24	Bacteroidetes	Saprospirae	[Saprospirales]	Chitinophagaceae	Sediminibacterium
525642	29	Bacteroidetes	Sphingobacteriia	Sphingobacteriales		
4461517	24	Bacteroidetes	Sphingobacteriia	Sphingobacteriales		
733904	24	Nitrospirae	Nitrospira	Nitrospirales		
716576	29	Planctomycetes	Planctomycetia	Rhodospirillales	Rhodospirillaceae	
356355	29	Planctomycetes	Planctomycetia	Planctomycetales	Planctomycetaceae	Planctomyces
832194	71	Proteobacteria	Alphaproteobacteria	Rhodospirillales	Rhodospirillaceae	
571360	65	Proteobacteria	Alphaproteobacteria	Rhodospirillales	Rhodospirillaceae	
569368	47	Proteobacteria	Alphaproteobacteria	Rhizobiales	Hyphomicrobiaceae	Rhodoplanes
756153	47	Proteobacteria	Alphaproteobacteria	Rhizobiales	Hyphomicrobiaceae	Rhodoplanes
560655	35	Proteobacteria	Alphaproteobacteria	Rickettsiales	Pelagibacteraceae	
574255	29	Proteobacteria	Alphaproteobacteria	Rhizobiales	Hyphomicrobiaceae	Hyphomicrobium
583130	29	Proteobacteria	Alphaproteobacteria	Rhodospirillales	Acetobacteraceae	
545287	24	Proteobacteria	Alphaproteobacteria			
923760	76	Proteobacteria	Betaproteobacteria			
593212	65	Proteobacteria	Betaproteobacteria			
203879	59	Proteobacteria	Betaproteobacteria			
548352	41	Proteobacteria	Betaproteobacteria	IS-44		
220761	35	Proteobacteria	Betaproteobacteria			
557255	29	Proteobacteria	Betaproteobacteria			
694781	29	Proteobacteria	Betaproteobacteria	MND1		
240278	29	Proteobacteria	Betaproteobacteria			
723936	29	Proteobacteria	Betaproteobacteria	Burkholderiales	Oxalobacteraceae	Polynucleobacter
977051	29	Proteobacteria	Betaproteobacteria	Methylophilales	Methylophilaceae	
536176	24	Proteobacteria	Betaproteobacteria	IS-44		
1109401	24	Proteobacteria	Betaproteobacteria			
593436	24	Proteobacteria	Betaproteobacteria	Gallionellales	Gallionellaceae	Gallionella
567333	24	Proteobacteria	Betaproteobacteria			
254098	24	Proteobacteria	Betaproteobacteria			
3984836	24	Proteobacteria	Betaproteobacteria			
771634	47	Proteobacteria	Deltaproteobacteria	Myxococcales	0319-6G20	

**Supplemental Data Sheet S10.** Core bacterial communities in the production well (n=10).						
**OTU**	**Detection Frequency %**	**Phylum**	**Class**	**Order**	**Family**	**Genus**
629448	20	Acidobacteria	Acidobacteria-5			
557143	70	Acidobacteria	Acidobacteria-6	iii1-15		
582670	50	Acidobacteria	Acidobacteria-6	iii1-15		
1111117	30	Acidobacteria	Acidobacteria-6	iii1-15		
138750	20	Acidobacteria	Acidobacteria-6	iii1-15		
179461	30	Bacteroidetes	Flavobacteriia	Flavobacteriales	Cryomorphaceae	Fluviicola
83335	30	Bacteroidetes	Sphingobacteriia	Sphingobacteriales		
525642	20	Bacteroidetes	Sphingobacteriia	Sphingobacteriales		
2775220	20	Bacteroidetes	Sphingobacteriia	Sphingobacteriales		
1139829	20	Crenarchaeota	Thaumarchaeota	Cenarchaeales	Cenarchaeaceae	Nitrosopumilus
159567	20	PAUC34f				
571360	100	Proteobacteria	Alphaproteobacteria	Rhodospirillales	Rhodospirillaceae	
583130	100	Proteobacteria	Alphaproteobacteria	Rhodospirillales	Acetobacteraceae	
832194	100	Proteobacteria	Alphaproteobacteria	Rhodospirillales	Rhodospirillaceae	
756153	80	Proteobacteria	Alphaproteobacteria	Rhizobiales	Hyphomicrobiaceae	Rhodoplanes
583185	60	Proteobacteria	Alphaproteobacteria	Rhizobiales	Hyphomicrobiaceae	
244655	50	Proteobacteria	Alphaproteobacteria	Rhodospirillales	Rhodospirillaceae	
956326	40	Proteobacteria	Alphaproteobacteria	Rhodospirillales	Rhodospirillaceae	
716576	30	Proteobacteria	Alphaproteobacteria	Rhodospirillales	Rhodospirillaceae	
353828	20	Proteobacteria	Alphaproteobacteria	Rhizobiales	Hyphomicrobiaceae	Rhodoplanes
3329856	20	Proteobacteria	Alphaproteobacteria	Rhodospirillales	Rhodospirillaceae	
1109401	100	Proteobacteria	Betaproteobacteria			
203879	100	Proteobacteria	Betaproteobacteria			
220761	100	Proteobacteria	Betaproteobacteria			
923760	80	Proteobacteria	Betaproteobacteria			
694781	70	Proteobacteria	Betaproteobacteria	MND1		
567333	60	Proteobacteria	Betaproteobacteria			
240278	60	Proteobacteria	Betaproteobacteria			
593212	60	Proteobacteria	Betaproteobacteria			
903951	30	Proteobacteria	Betaproteobacteria			
4415049	30	Proteobacteria	Betaproteobacteria			
224230	30	Proteobacteria	Betaproteobacteria			
254098	30	Proteobacteria	Betaproteobacteria			
817446	30	Proteobacteria	Betaproteobacteria			
1110386	30	Proteobacteria	Betaproteobacteria			
536176	20	Proteobacteria	Betaproteobacteria	IS-44		
205810	20	Proteobacteria	Betaproteobacteria			
508906	20	Proteobacteria	Betaproteobacteria			
3984836	20	Proteobacteria	Betaproteobacteria			
1112928	20	Proteobacteria	Deltaproteobacteria	Syntrophobacterales	Syntrophobacteraceae	
